# Penetration of CdSe/ZnS quantum dots into differentiated vs undifferentiated Caco-2 cells

**DOI:** 10.1186/s12951-016-0222-9

**Published:** 2016-09-26

**Authors:** Henrike Peuschel, Thomas Ruckelshausen, Silke Kiefer, Yuliya Silina, Annette Kraegeloh

**Affiliations:** INM - Leibniz Institute for New Materials, Campus D2 2, 66123 Saarbrücken, Germany

**Keywords:** Quantum dots, Human intestinal cells (Caco-2), Penetration depth, Differentiation

## Abstract

**Background:**

Quantum dots (QDs) have great potential as fluorescent labels but cytotoxicity relating to extra- and intracellular degradation in biological systems has to be addressed prior to biomedical applications. In this study, human intestinal cells (Caco-2) grown on transwell membranes were used to study penetration depth, intracellular localization, translocation and cytotoxicity of CdSe/ZnS QDs with amino and carboxyl surface modifications. The focus of this study was to compare the penetration depth of QDs in differentiated vs undifferentiated cells using confocal microscopy and image processing.

**Results:**

Caco-2 cells were exposed to QDs with amino (NH_2_) and carboxyl (COOH) surface groups for 3 days using a concentration of 45 µg cadmium ml^−1^. Image analysis of confocal/multiphoton microscopy z-stacks revealed no penetration of QDs into the cell lumen of differentiated Caco-2 cells. Interestingly, translocation of cadmium ions onto the basolateral side of differentiated monolayers was observed using high resolution inductively coupled plasma mass spectrometry (ICP-MS). Membrane damage was neither detected after short nor long term incubation in Caco-2 cells. On the other hand, intracellular localization of QDs after exposure to undifferentiated cells was observed and QDs were partially located within lysosomes.

**Conclusions:**

In differentiated Caco-2 monolayers, representing a model for small intestinal enterocytes, no penetration of amino and carboxyl functionalized CdSe/ZnS QDs into the cell lumen was detected using microscopy analysis and image processing. In contrast, translocation of cadmium ions onto the basolateral side could be detected using ICP-MS. However, even after long term incubation, the integrity of the cell monolayer was not impaired and no cytotoxic effects could be detected. In undifferentiated Caco-2 cells, both QD modifications could be found in the cell lumen. Only to some extend, QDs were localized in endosomes or lysosomes in these cells. The results indicate that the differentiation status of Caco-2 cells is an important factor in internalization and localization studies using Caco-2 cells. Furthermore, a combination of microscopy analysis and sensitive detection techniques like ICP-MS are necessary for studying the interaction of cadmium containing QDs with cells.

**Electronic supplementary material:**

The online version of this article (doi:10.1186/s12951-016-0222-9) contains supplementary material, which is available to authorized users.

## Background

Nanotechnological applications using quantum dots (QDs) have great potential especially in the biomedical field where high photostability and narrow emission spectra render them good candidates for fluorescent labels in cell tracking studies or for multiplex imaging [1]. Besides using QD nanocrystals with their unique luminescent properties for bio-imaging, QD based nanosensors have proven to be valid detectors of cancer biomarkers [2, 3]. Prior to use of QDs in biomedical applications, knowledge about degradation, uptake efficiency, cellular localization, translocation and cytotoxicity is essential in order to design safe particles. Knowledge about potential adverse effects derives from various in vitro studies in which cytotoxic effects induced by cadmium selenide (CdSe) QDs could be correlated with the release of Cd^2+^ ions [4, 5], while additional passivation of the CdSe core with a semiconductor shell (ZnS) was observed to prevent the release of Cd^2+^ ions and decreased cytotoxic effects in vitro [4, 6]. This shows that the surface chemistry of QDs has a great influence on cytotoxic effects.

Toxicity studies indicate the production of reactive oxygen species (ROS) after exposure to QDs, which can cause cell damage and death and modulate signaling processes [7, 8]. Furthermore, cellular uptake of QDs was reported in various in vitro studies, which will raise the probability of long term cytotoxic effects in tissues. Uptake via endocytosis and subcellular localization of QDs in early endosomes and lysosomes was shown for different cell lines [9–11].

Cellular uptake of QDs depends on the surface modification. Zhang and Monteiro-Riviere investigated the mechanisms of cellular uptake for CdSe/ZnS QDs functionalized with different surface coatings and observed that carboxylic coated QDs were taken up in greater amounts compared to PEG-amine coated QDs [11]. After particle internalization, QDs are reported to localize in the perinuclear region [6, 11], while no particles were found in the nucleus [12]. As particles are localized in lysosomes where low pH values are present, which influences the QD stability [5], prevention of degradation is especially important if particles come in contact with acidic environments. Loginova et al. reported that a combination of polythiol ligands and silica shell around CdSe/ZnS QDs prevent the degradation and fluorescence quenching after oral administration in the gastrointestinal tract of mice [13].

Studying the interaction of QDs with intestine cells is essential. Especially the presence of QDs in the environment and subsequently in drinking water or the food chain might rise in the future due to increase usage in various applications. Therefore, toxicological studies on this field are required. As a cell model to mimic the intestine barrier and absorptive enterocytes in pharmacological and toxicological studies, Caco-2 cells differentiated on transwell membranes are used providing access to apical as well as basolateral epithelia. A concentration-dependent uptake of cadmium (Cd) in differentiated Caco-2 cells was reported, while only slow transepithelial transport was observed and no effect on the integrity of the cell membrane or the transepithelial resistance (TER) [14]. However, information is lacking on how efficiently functionalized CdSe/ZnS QDs penetrate into differentiated Caco-2 cell monolayers in comparison to undifferentiated Caco-2 cells, which are often used in uptake and cytotoxicity studies. Therefore, in the present study, we investigated the penetration depth, localization and cytotoxicity of carboxyl- and amino- functionalized CdSe/ZnS QDs in Caco-2 cells. Caco-2 cells cultured on transwell membranes for differentiation were used to obtain a uniform monolayer. The translocation of cadmium from the apical epithelium onto the basolateral side was investigated after long term exposure to QDs at a concentration of 45 µg cadmium ml^−1^ for 3 days using inductively coupled plasma mass spectrometry (ICP-MS). The utilized cadmium concentration is below the concentrations that were chosen in in vivo studies for imaging [15]. However, studies of long term effects using lower concentrations which are interesting when thinking about repeated dosing and accumulation of particles after in vivo applications of high doses are necessary in order to design safe particles.The dependency of the differentiation status of Caco-2 cells and functionalization of QDs on the penetration depth was investigated using confocal and two photon microscopy. In addition, membrane integrity was tested and generation of reactive oxygen species was measured.

## Results

### Physico-chemical properties of QDs

**Fig. 1:**
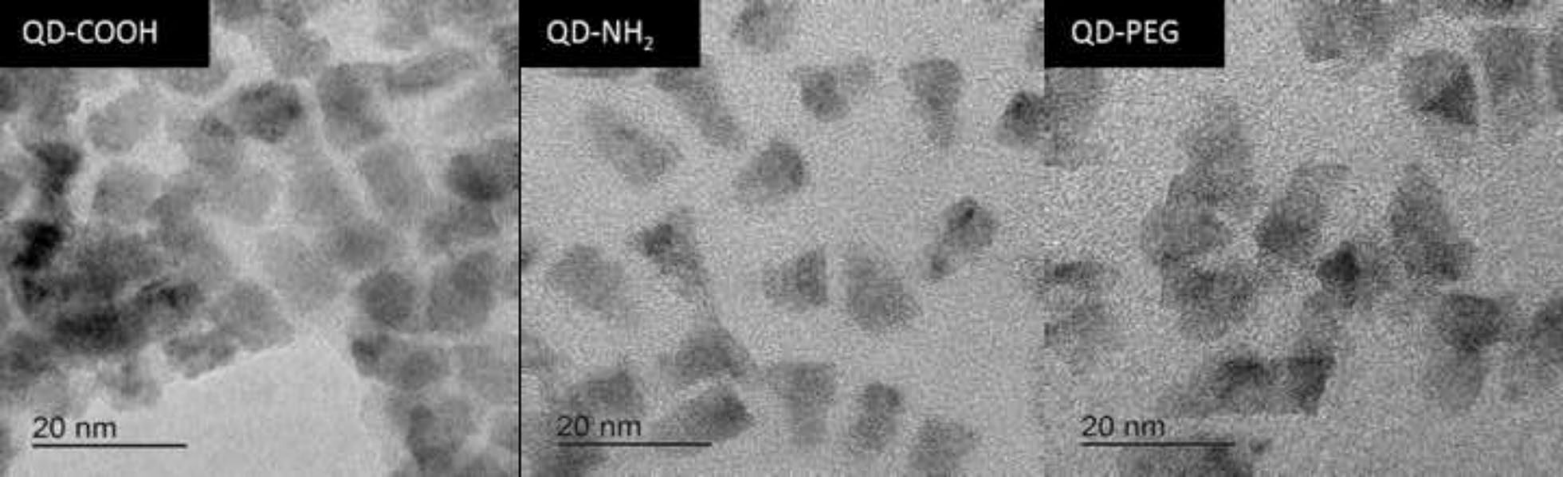
Electron micrographs of QDs with different surface coatings

Prior to use of CdSe/ZnS QDs in cell experiments, size and morphology, zeta potential and hydrodynamic diameter of all functionalized particles were determined (Table 1). QDs appeared as elongated, roughly cone-shaped structures. The height of the QDs was determined from electron micrographs (Fig. 1) to be 13 nm for QD-COOH and QD-NH_2_ and 14 nm for QD-PEG, respectively. The height of the QDs was uniform with variations below 8 %, indicating a narrow size distribution of the particle. The presence of lattice planes at higher magnification (data not shown) indicated crystallinity of the QDs. The hydrodynamic diameter of QD-COOH dispersed in water and DPBS was found to be comparable to the corresponding size determined by transmission electron microscopy (TEM). The mean hydrodynamic diameter of QD-NH_2_ and QD-PEG dispersed in water could not be determined due to a broad size distribution. After dispersion in DPBS, the hydrodynamic diameter of QD-PEG was two-fold larger than the one of QD-COOH, which might be explained by the presence of a bulky PEG linker coupled to an amphiphilic polymer absent from the QD-COOH coating. The hydrodynamic diameter of QDs dispersed in cell culture medium containing 20 % serum (MEM+) was apparently dominated by the presence of a more than ten-fold excess of protein molecules over nanoparticles (approximately 0.1 M BSA compared with 8 µM QDs). The measured values therefore rather represent the hydrodynamic diameter of the proteins contained in the medium. Pure cell culture medium without QDs was measured as a reference and hydrodynamic diameters of 7 ± 2 nm were obtained representing the size of BSA, which is the most abundant protein in the medium (data not shown).

**Table 1:** Physico-chemical properties of QDs used in the study

Sample	Hydrodynamic diameter [nm]			Height (TEM) [nm]	Zeta potential [mV]		
	Water	DPBS	MEM+		Water	DPBS	MEM+
QD-COOH	17 ± 4 (21 %)	19 ± 4 (22 %)	10 ± 4 (37 %)^a^	13 ± 1	−39 ± 3	−36 ± 2	−8 ± 3
QD-NH_2_	ND	ND	34 ± 18 (53 %)	13 ± 1	−36 ± 1	−2 ± 0	−3 ± 1
QD-PEG	ND	38 ± 10 (25 %)	11 ± 4 (41 %)^a^	14 ± 1	−25 ± 0	0 ± 2	−3 ± 1

^a^ Pure cell culture medium (MEM+) without QDs was measured as a reference and hydrodynamic diameters of 7 ± 2 nm were obtained representing the size of BSA, which is the most abundant protein in MEM+.The measured values therefore rather represent the hydrodynamic diameter of the proteins in the medium

All particles exhibited a negative zeta potential when dispersed in water, ranking from −39 to −25 mV (Table 1). After dispersion in DPBS, QD-COOH, which are stabilized electrostatically by a high number of COO^−^ groups on the particle surface (provider information), still exhibited a zeta potential of −36 mV, indicating stability of the particles. Under comparable conditions, the zeta potential values of QD-NH_2_ and QD-PEG were significantly reduced, indicating neutralization of charges on the particle surface. Nevertheless, both QD-PEG and QD-NH_2_ are described (provider information) to be stabilized sterically by PEG anchored to the particle surfaces. The zeta potential values of all particles in MEM+ were again dominated by the zeta potential of the proteins contained in serum. As specified by the distributor, the molar particle concentration of undiluted QD dispersions were 2 µM (QD-PEG) and 8 µM (QD-COOH, QD-NH_2_). As determined by ICP-OES, the cadmium concentration of the latter particle stocks was 4589 ± 0.05 µg ml^−1^ for QD-NH_2_ and 6551 ± 0.02 µg ml^−1^ for QD-COOH. Experiments were performed in presence of either an equal Cd concentration (45 µg Cd ml^−1^, corresponding to 56 nM QD-COOH and 80 nM QD-NH_2_ and 22 µg Cd ml^−1^, corresponding to 48 nM QD-NH_2_) or an equal molar particle concentration of 16 nM.

### Fluorescence stability in cell culture medium

**Fig. 2:**
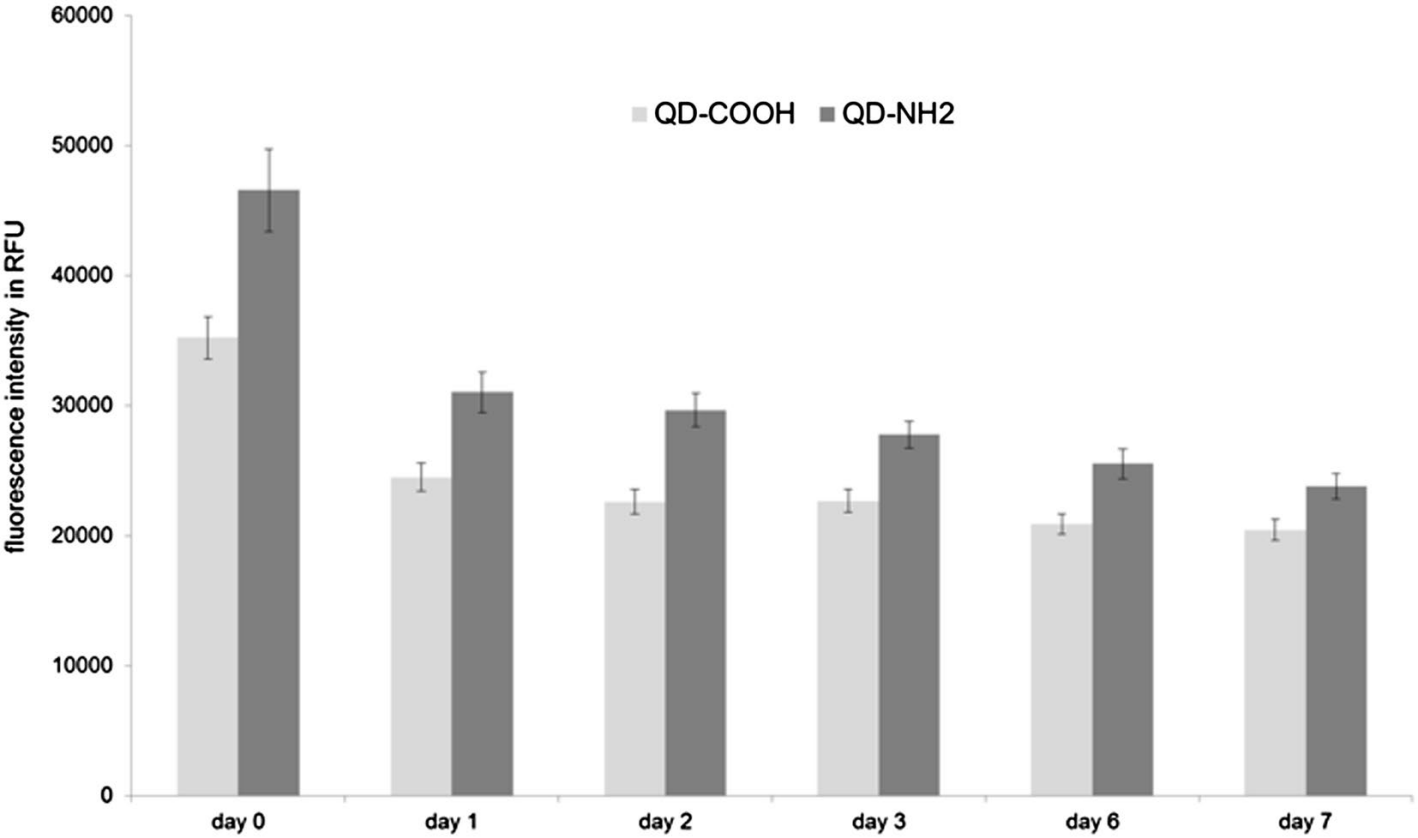
Fluorescence stability of QDs. Fluorescence intensity of QD-COOH and QD-NH_2_ dispersed in cell culture medium was measured for up to 7 days. Excitation wavelength was 380 nm. Emission was measured at 655 nm

Fluorescence stability of QD dispersions (45 µg cadmium ml^−1^) in cell culture medium was measured for up to 7 days at an excitation wavelength of 380 nm. QD-NH_2_ had a higher fluorescence (46552 RFU) when compared to QD-COOH (35193 RFU), which derives from different ligands on the particle surface and different agglomeration behavior in cell medium. But both QD types showed a similar trend of decreasing fluorescence intensity over time (Fig. 2). After 24 h, 30 % reduction of the fluorescence intensity was measured. Within 3 days, no further decrease of fluorescence values was found. Therefore, experiments were performed within this time frame. Longer incubation times revealed a further decrease in fluorescence intensity. After 7 days a loss of almost 50 % compared to the initial fluorescence intensity was observed.

### Cellular internalization

Caco-2 cells grown on transwell membranes were used to investigate the internalization of QDs. Initial experiments were performed at a molar particle concentration of 16 nM, but rarely emission signals related to QDs were detected. Especially QD-PEG were hard to detect at all on or in Caco-2 cells (Additional files 1, 2, 3), although they exhibited a similar fluorescence intensity compared to QD-COOH and QD-NH_2_. Therefore, only QD-COOH and QD-NH_2_ were used for internalization studies at a concentration of 45 µg cadmium ml^−1^. Cells were exposed to QDs for 3 days, prior to 3D imaging. At the higher concentration, more QD related emission signals were detected.

**Fig. 3:**
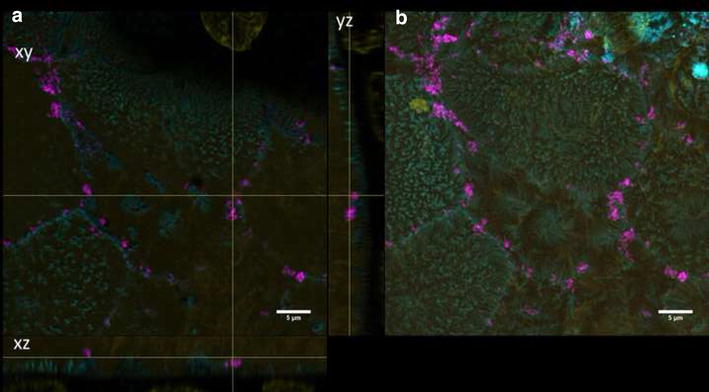
Differentiated cells exposed to QD-COOH. Confocal images of differentiated Caco-2 cells incubated for 3 days with QD-COOH (45 µg cadmium ml^−1^). **a** Orthogonal views (xy, xz and yz) showing the intersection planes at the position of the yellow cross-hair. **b** Maximum intensity projection of the same z-stack. QDs (*magenta*), cell membrane (*cyan*) and nucleus (*yellow*)

**Fig. 4:**
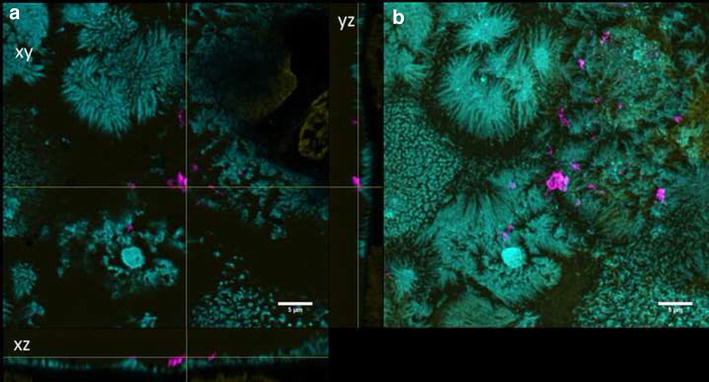
Differentiated cells exposed to QD-NH_2_. Confocal images of differentiated Caco-2 cells incubated for 3 days with QD-NH_2_ (45 µg cadmium ml^−1^). **a** Orthogonal views (xy, xz and yz) showing the intersection planes at the position of the yellow cross-hair. **b** Maximum intensity projection of the same z-stack. QDs (*magenta*), cell membrane (*cyan*) and nucleus (*yellow*)

For differentiation, cells were grown on transwell membranes for 21 days. These differentiated cells exhibited microvilli structures on the apical side as revealed by SEM micrographs. TER values higher than 250 Ω/cm^2^ were measured representing intact monolayers. Furthermore, ZO-1 protein was expressed, which plays an important role in the formation of tight junctions. In differentiated cells, both QD modifications could mainly be detected on the cell surfaces and barely inside the lumen (Figs. 3, 4). No change in TER after 3 days exposure of differentiated Caco-2 layers to QDs was observed (data not shown).

**Fig. 5:**
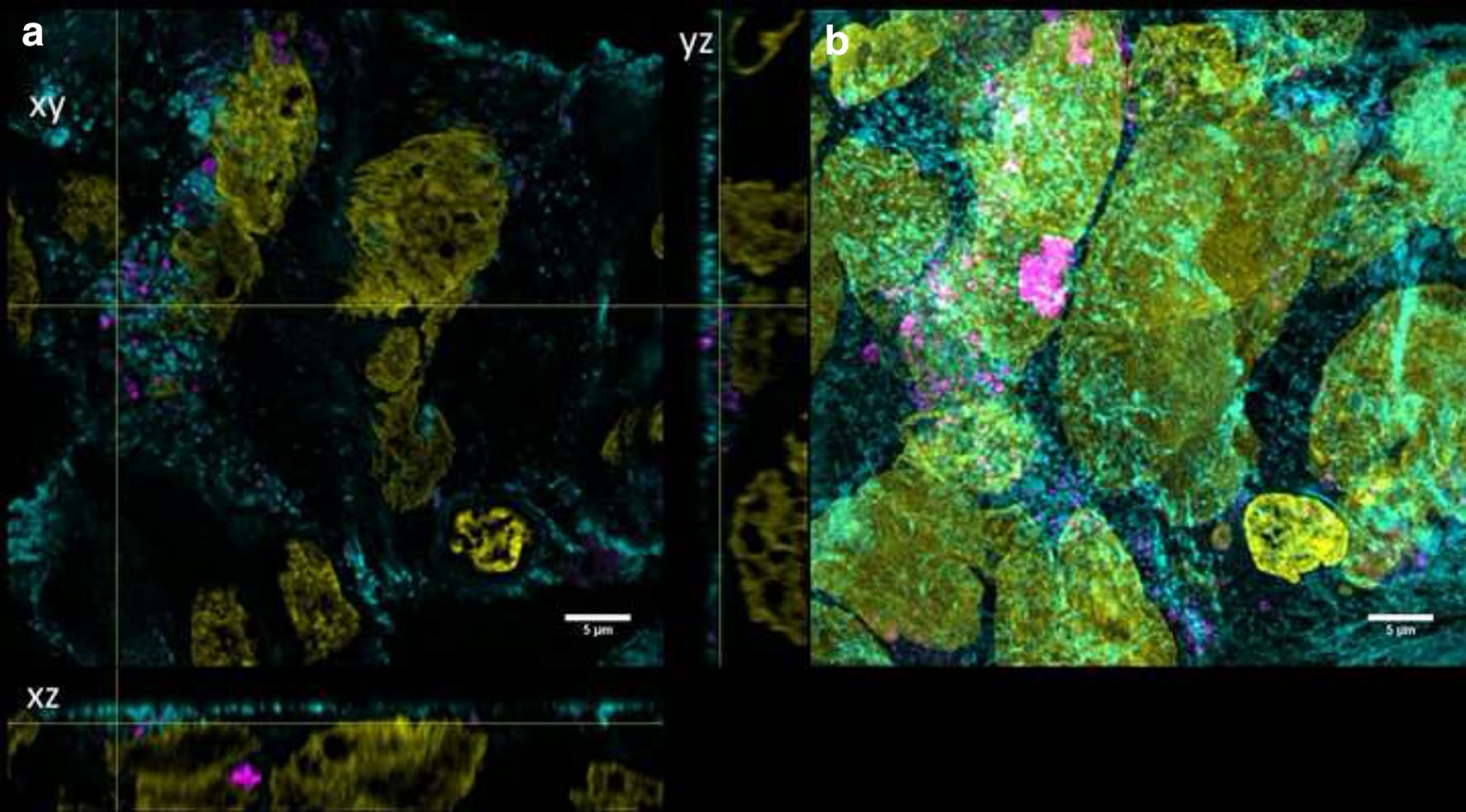
Undifferentiated cells exposed to QD-COOH. Confocal images of undifferentiated Caco-2 cells incubated for 3 days with QD-COOH (45 µg cadmium ml^−1^). **a** Orthogonal views (xy, xz and yz) showing the intersection planes at the position of the yellow cross-hair. **b** Maximum intensity projection of the same z-stack. QDs (*magenta*), cell membrane (*cyan*) and nucleus (*yellow*)

**Fig. 6:**
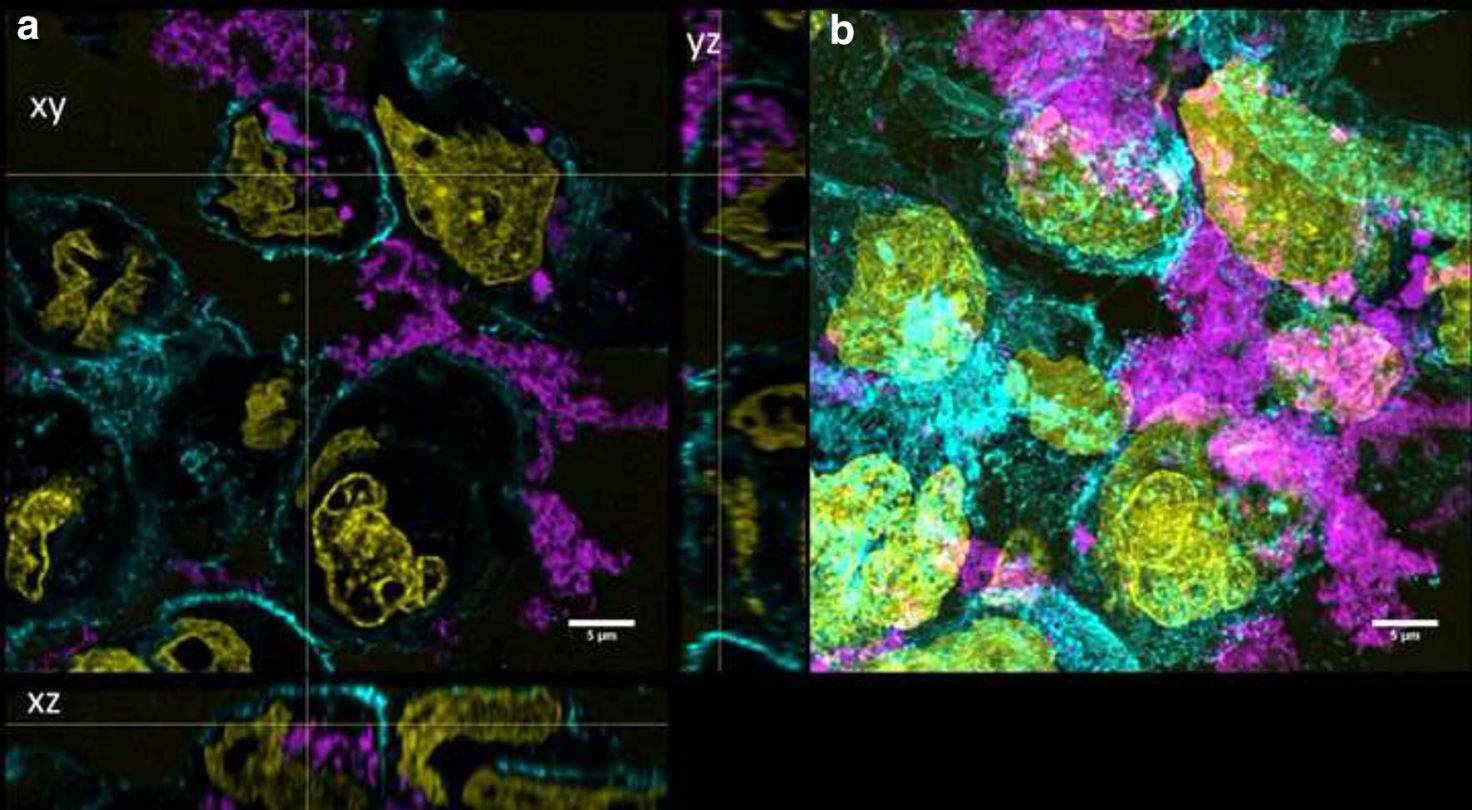
Undifferentiated cells exposed to QD-NH_2_. Confocal images of undifferentiated Caco-2 cells incubated for 3 days with QD-NH_2_ (45 µg cadmium ml^−1^). **a** Orthogonal views (xy, xz and yz) showing the intersection planes at the position of the yellow cross-hair. **b** Maximum intensity projection of the same z-stack. QDs (*magenta*), cell membrane (*cyan*) and nucleus (*yellow*)

Undifferentiated Caco-2 cells were grown for 2 days on transwell membranes and showed no expression of ZO-1 and low TER values (data not shown). QD-COOH appeared to be associated with the cell membrane of undifferentiated cells and formed agglomerates (Fig. 5b). Orthogonal slices revealed that QDs were also present within the cell lumen (Fig. 5a). QD-NH_2_ formed even larger agglomerates on the cell surface of undifferentiated Caco-2 cells, which were also detected within the cell lumen (Fig. 6).

Control images showed the same morphology and number of cells per area compared to treated Caco-2 cells (Additional files 4 and 5).

### Penetration depth analysis

**Fig. 7:**
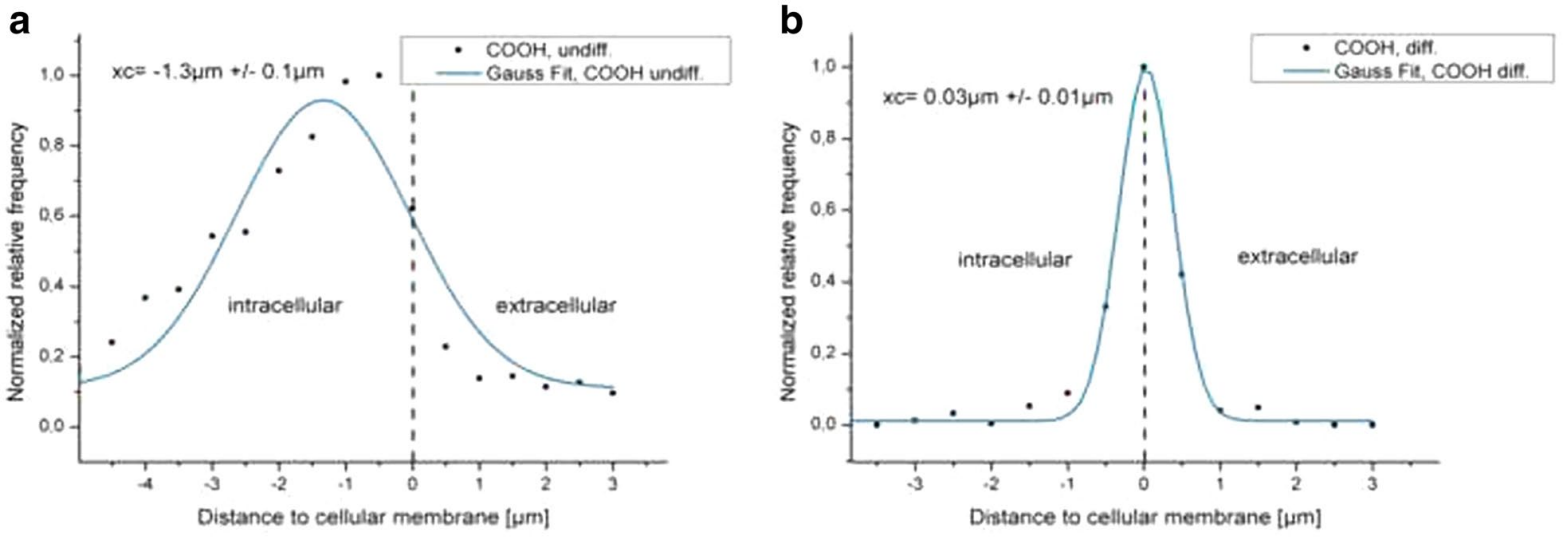
Distances of QD-COOH to the cytoplasmic membrane. Histograms of distances between the membrane and QD-COOH in undifferentiated cells (**a**) and in differentiated cells (**b**) using a concentration of 45 µg cadmium ml^−1^. X_c_ represents the peak position and standard deviation of the Gauss fit

**Fig. 8:**
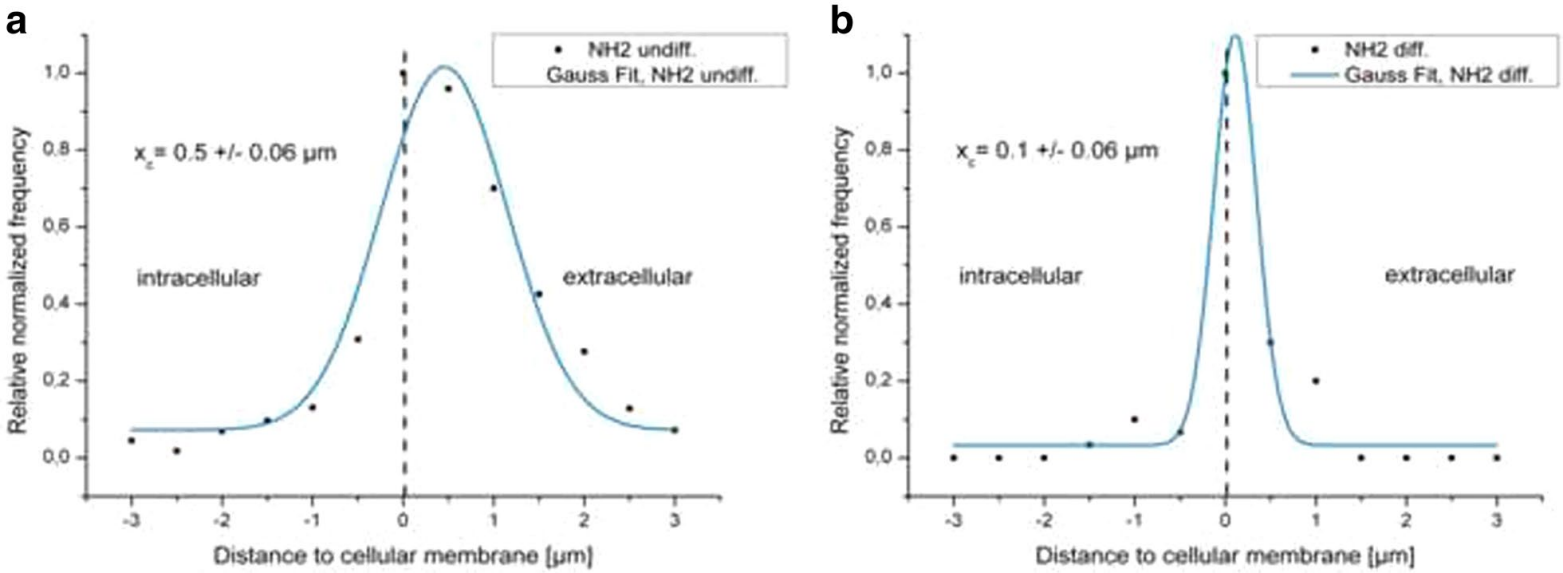
Distances of QD-NH_2_ to the cytoplasmic membrane. Histograms of distances between the membrane and QD-NH_2_ in undifferentiated cells (**a**) and in differentiated cells (**b**) using a concentration of 22 µg cadmium ml^−1^. X_c_ represents the peak position and standard deviation of the Gauss fit

At least 10 z-stacks out of three independent experiments were used for the analysis of the penetration depth. Images were deconvolved and segmented prior to distance measurements to reveal the penetration depth of the QDs in differentiated or undifferentiated Caco-2 cells. For estimation of the mean distance, results were plotted and fitted. Gaussian fit was adopted in case of a Gaussian distribution. QD-COOH penetrated into the cell lumen of undifferentiated Caco-2 cells (Fig. 7a). The maximum of the Gauss fit revealed a mean distance from the cellular membrane of −1.3 ± 0.1 µm indicating penetration into the cell lumen. On the other hand, no penetration of QD-COOH into differentiated cells was detected and a mean distance value of 0.03 ± 0.01 µm was measured between QD-COOH and the membrane of differentiated cells, indicating that particles only associate with the membrane and do not significantly penetrate into the cell lumen (Fig. 7b). Therefore, image analysis confirmed the aforementioned observations made on the basis of visual inspection of the microscopy images. As shown in Fig. 6, QD-NH_2_ could be detected in undifferentiated cells, but at a concentration of 45 µg cadmium ml^−1^, the QDs formed large agglomerates, which could not be separated via the watershed algorithm anymore. Since an analysis of the penetration depth would have led to biased results in this case, we decided to use a two-fold lower cadmium concentration of QD-NH_2_ (22 µg cadmium ml^−1^) for the penetration depth analysis. Interestingly, no penetration of QD-NH_2_ into undifferentiated as well as differentiated cells was measured (Fig. 8). QD-NH_2_ particles were mainly detected on the cell surface as indicated by the mean distance of 0.5 ± 0.06 µm Fig. 8a and 0.1 ± 0.06 µm, respectively Fig. 8b.

### Intracellular localization

To determine the intracellular localization of QDs in Caco-2 cells, early endosomes and lysosomes were immunostained. Even after these long incubation time, colocalization with these vesicles was assumed, because they are constantly formed. In differentiated Caco-2 cells, internalization was a rare event as described before and therefore, no particles could be detected in lysosomes (data not shown).

**Fig. 9:**
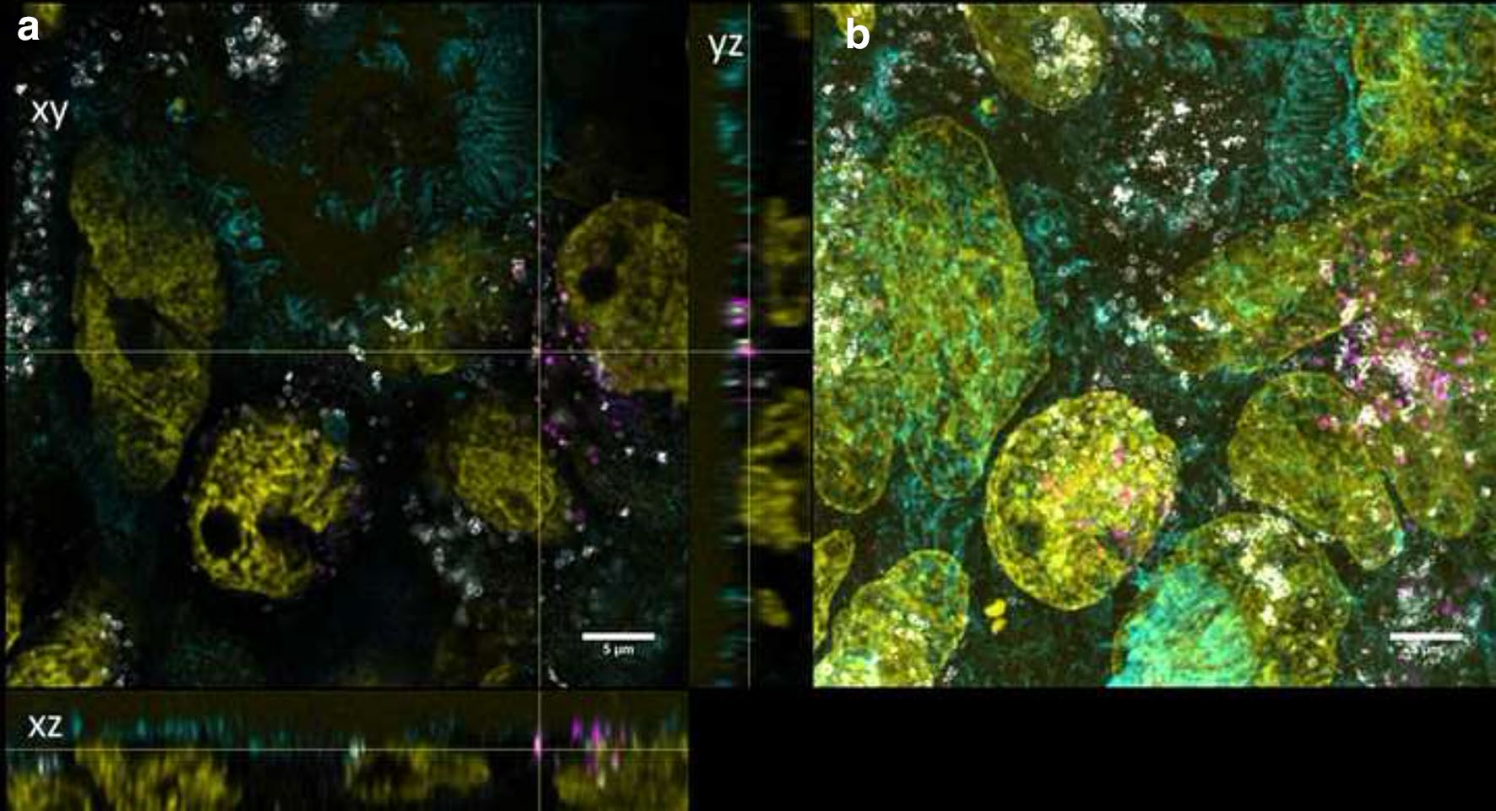
Intracellular localization of QD-COOH in undifferentiated Caco-2 cells. Confocal images of undifferentiated Caco-2 cells incubated for 3 days with QD-COOH (45 µg cadmium ml^−1^). Early endosomes (EEA1) are depicted in *grey*. **a** Orthogonal views (*xy*, *xz* and *yz*) showing the intersection planes at the position of the yellow cross-hair. **b** Maximum intensity projection of the same z-stack. QDs (*magenta*), cell membrane (*cyan*), and nucleus (*yellow*)

**Fig. 10:**
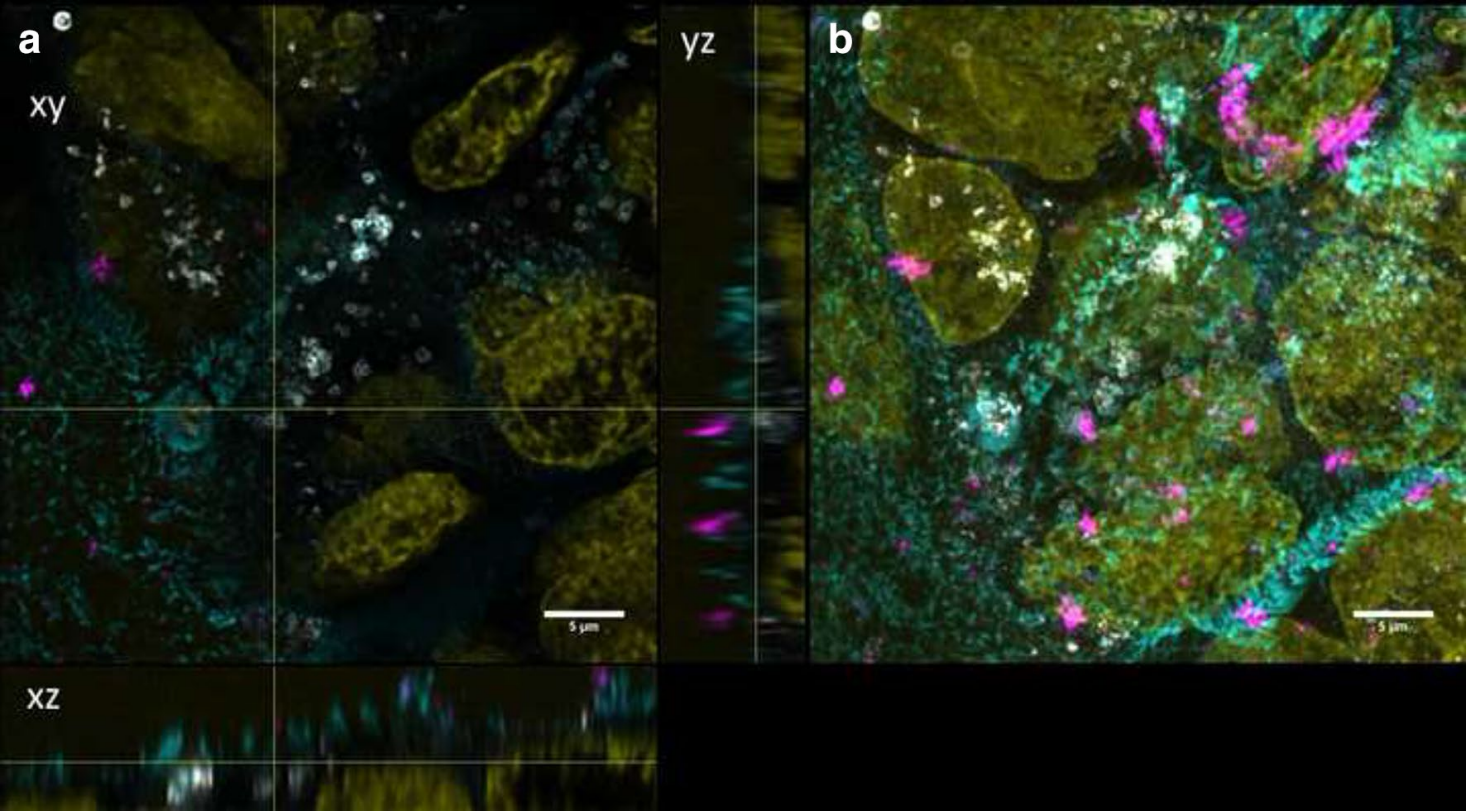
Intracellular localization of QD-NH_2_ in undifferentiated Caco-2 cells. Confocal images of undifferentiated Caco-2 cells incubated for 3 days with QD-NH_2_ (45 µg cadmium ml^−1^). Early endosomes (EEA1) are depicted in *grey* **a**. Orthogonal views (*xy*, *xz* and *yz*) showing the intersection planes at the position of the yellow cross-hair. **b** Maximum intensity projection of the same z-stack. QDs (*magenta*), cell membrane (*cyan*), and nucleus (*yellow*)

**Fig. 11:**
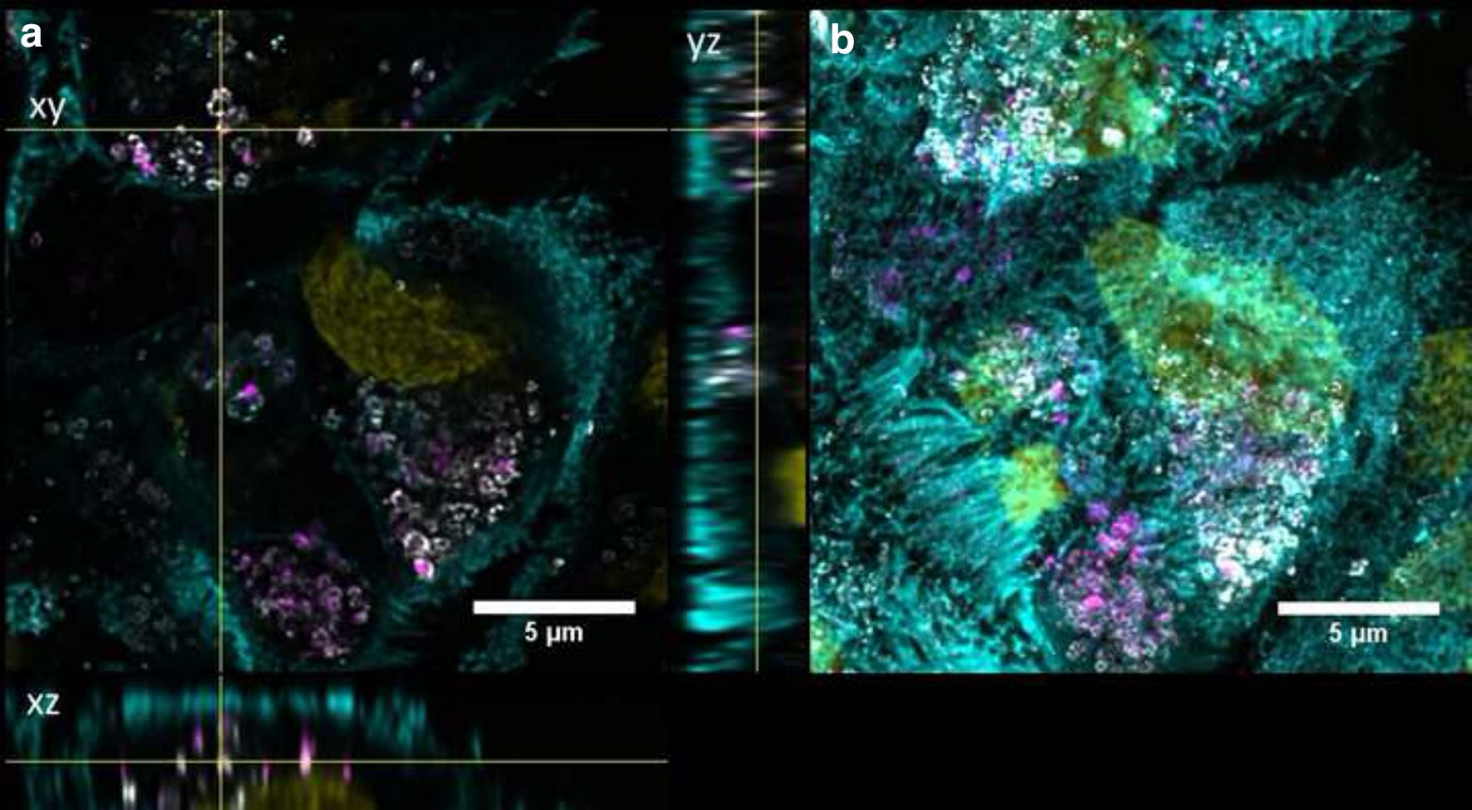
Intracellular localization of QD-COOH in undifferentiated Caco-2 cells. Confocal images of undifferentiated Caco-2 cells incubated for 3 days with QD-COOH (45 µg cadmium ml^−1^). Lysosomes (Lamp1) are depicted in *grey*. **a** Orthogonal views (*xy*, *xz* and *yz*) showing the intersection planes at the position of the yellow cross-hair. **b** Maximum intensity projection of the same z-stack. QDs (*magenta*), cell membrane (*cyan*), and nucleus (*yellow*)

**Fig. 12:**
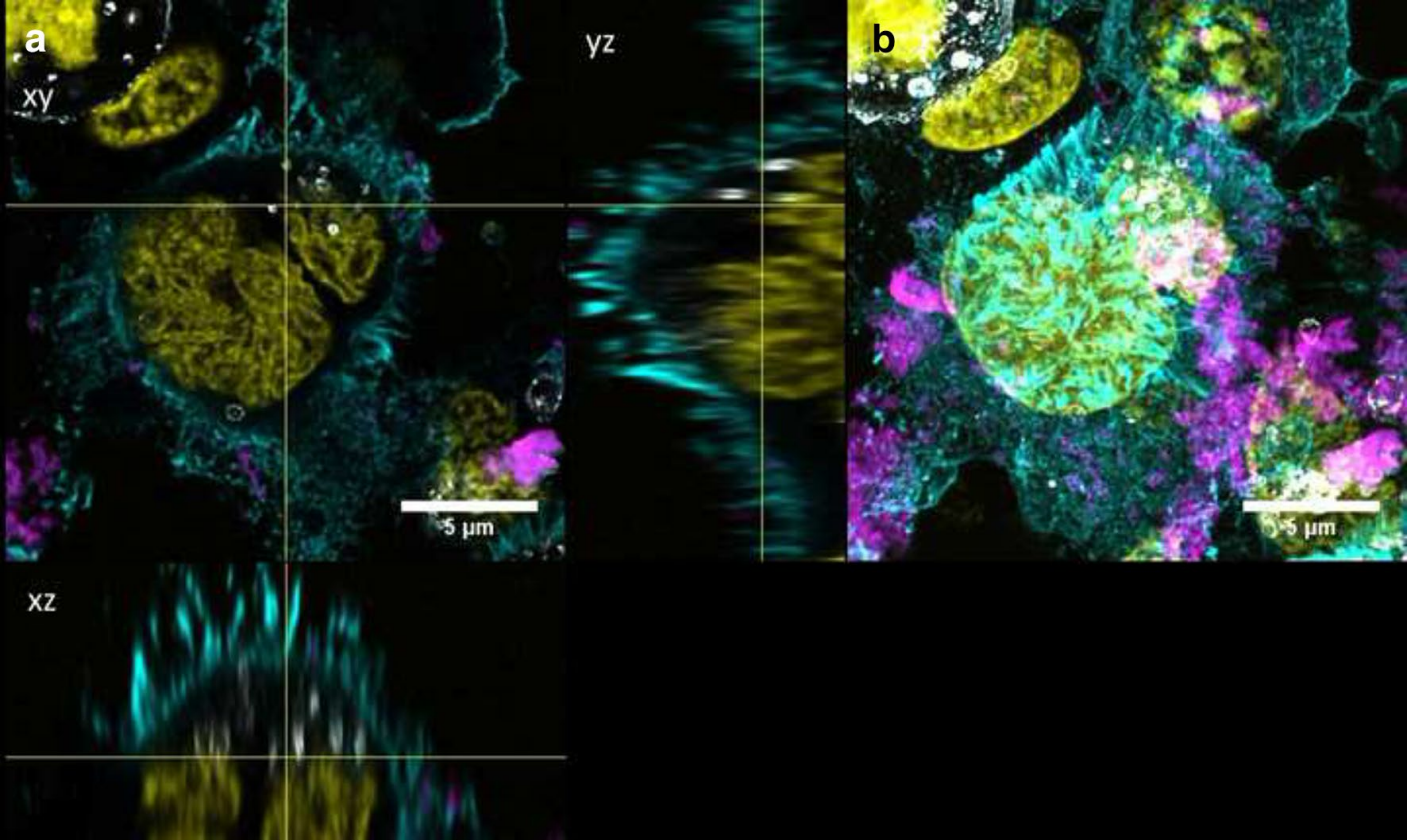
Intracellular localization of QD-NH_2_ in undifferentiated Caco-2 cells. Confocal images of undifferentiated Caco-2 cells incubated for 3 days with QD-NH_2_ (45 µg cadmium ml^−1^). Lysosomes (Lamp1) are depicted in *grey*. **a** Orthogonal views (*xy*, *xz* and *yz*) showing the intersection planes at the position of the yellow cross-hair. **b** Maximum intensity projection of the same z-stack. QDs (*magenta*), cell membrane (*cyan*), and nucleus (*yellow*)

In undifferentiated cells, evaluation of image stacks did not indicate colocalization of QD-NH_2_ (Fig. 9) with early endosomes. For QD-COOH, colocalization with early endosomes after 3 days incubation was a rare event as well in these cells (Fig. 10). Here, the mean diameter of EEA-1 positive endosomes was measured to be 0.6 ± 0.1 µm. On the other hand, colocalization with lysosomes was observed for a small amount of QD-COOH (Fig. 11), suggesting that a part of these particles finally localize in these organelles after 3 days incubation. For QD-NH_2_, colocalization with lysosomes was a rare event as observed in microscopy images (Fig. 12). The mean diameter of lamp-1 positive lysosomes was measured to be 0.9 ± 0.5 µm, thus larger compared to early endosomes.

### Cytotoxicity of QDs

**Fig. 13:**
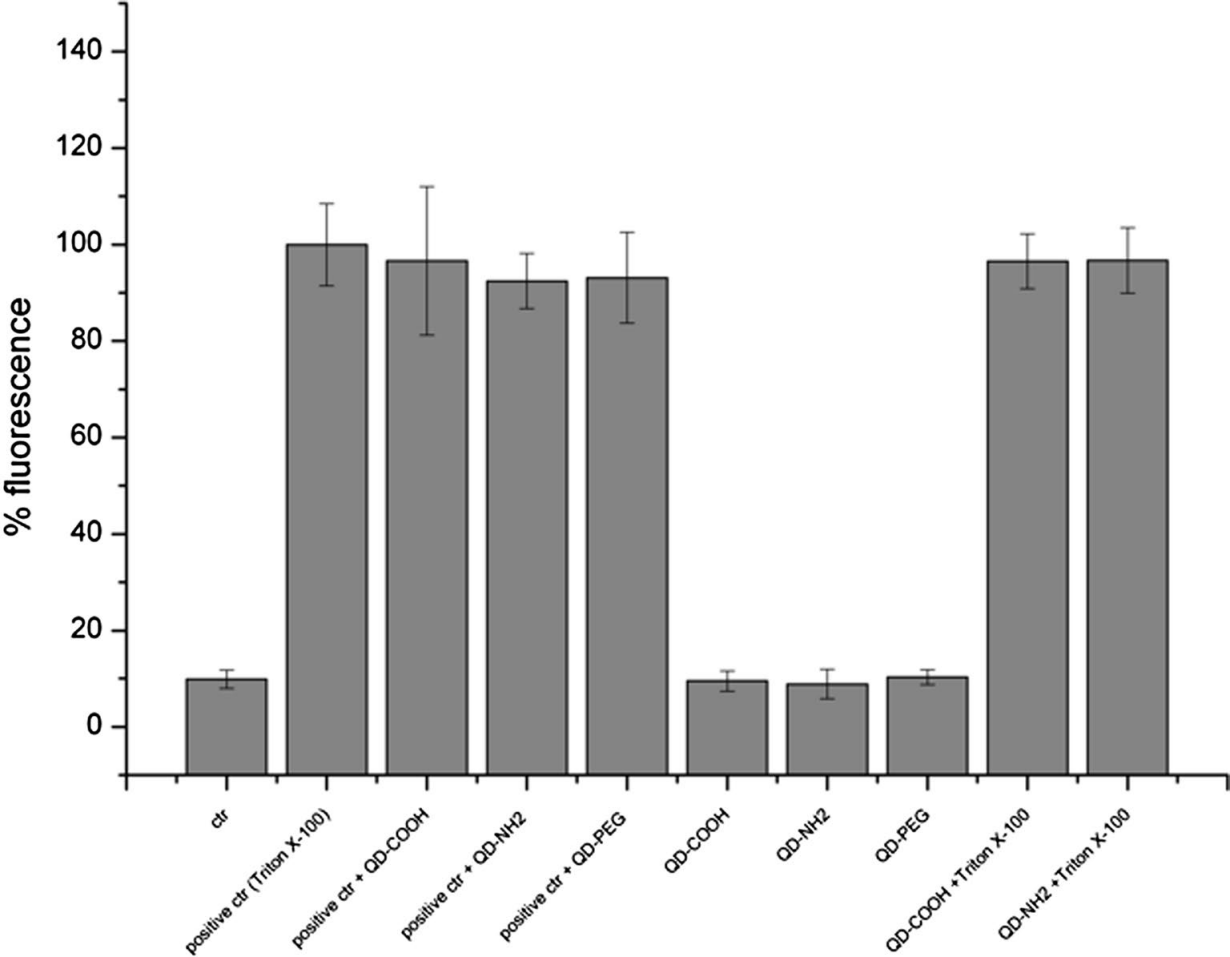
Membrane integrity measurements using CellTox™ Green Assay. Membrane integrity was measured after 3 days exposure of undifferentiated cells to QD-COOH, QD-NH_2_ and QD-PEG (45 µg cadmium ml^−1^). Interference with the assay was tested by addition of QDs to positive control Triton X-100 (positive ctr + QDs) shortly prior fluorescence measurement or by adding Triton X-100 to cells exposed to QDs for 3 days (QD + Triton X-100)

As QDs were associated with the membrane, membrane integrity of undifferentiated Caco-2 cells was investigated using the non-enzyme assay (CellTox™ Green). Even at a concentration of 45 µg cadmium ml^−1^, no membrane damage was induced by QD-COOH and QD-NH_2_ after 3 days incubation (Fig. 13). No interference with the fluorescence signals of 0.2 % Triton X-100 lysed cells induced by QDs was detected. On the other hand, interference with enzyme assays was detected using CytoTox-ONE™. Here, controls showed a significant decrease in fluorescence signals of Triton X-100 lysed cells after addition of QDs (Additional file 6). Interference was also detected using the H_2_DCF-DA assay for measurement of ROS. The fluorescence signals of cells incubated in the presence of the positive control with QDs showed significantly increased values (Additional file 7).

### Transepithelial transport of Cd

To investigate if QDs, when added apically, are able to pass the cell-layer and the transwell membrane (ThinCert) to reach the lower well and therefore the basolateral side of the cells, the cadmium concentration was determined in cell-culture medium of the lower well 3 days after addition of QDs at a cadmium concentration of 45 µg ml^−1^. The TER values of the same samples in which the cadmium transport was measured were in the same range (280 ± 36 Ω/cm^2^ for cells incubated with QD-COOH, and 317 ± 35 Ω/cm^2^ for cells incubated with QD-NH_2_).

**Fig. 14:**
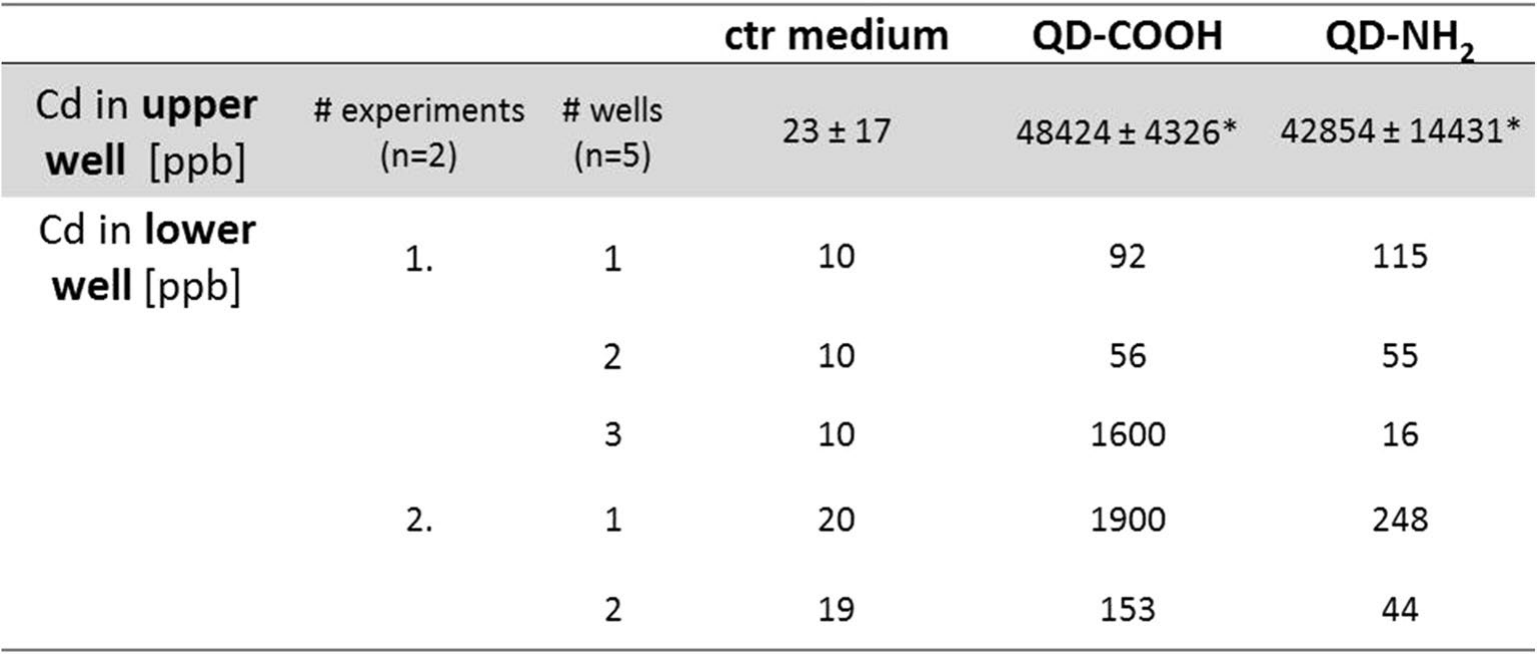
Transepithelial transport of cadmium. Differentiated Caco-2 cells were incubated for 3 days with QD-COOH or QD-NH_2_ using a concentration of 45 µg cadmium ml^−1^. Cadmium translocation into the lower well was measured using ICP-MS. The precision of the assay was $\le$ 5 % RSD (n = 5) and method accuracies were between 93.6 and 108.7 %. *Cadmium retrieval in ThinCert™ after 3 days exposure was measured using ICP-OES

The background cadmium concentration in medium was in the same range in both upper and lower well (23 ± 17 and 14 ± 5 ppb). The Cd concentration in the lower wells of Caco-2 cells exposed to QD-COOH was significantly higher compared to the untreated control (Fig. 14). There was a high variance in detected Cd concentrations between individual wells of two independent experiments and concentrations from 92 up to 1900 ppb Cd were measured. After exposure to QD-NH_2_, Cd concentrations from 16 to 248 ppb were measured in the lower well. The retrieval of cadmium in the upper well after incubation was 48424 ± 4326 ppb (48 ± 4 µg ml^−1^) for QD-COOH and 42854 ± 14431 ppb (43 ± 14 µg ml^−1^) for QD-NH_2_ which was detected using ICP-OES. Thus, only 0.1–4 % of the applied Cd either in form of QDs or in ionic form reached the lower compartment. In control experiments a passage of cadmium across 3 µm pore-sized membranes without Caco-2 cells of 20 % and an equal distribution between upper and lower well was observed after 3 days exposure to 45 µg ml^−1^QD-COOH (data not shown).

## Discussion

The aim of the present study was to investigate the penetration depth and intracellular localization of CdSe/ZnS QDs functionalized with COOH or NH_2_ with special emphasis on the differentiation status of Caco-2 cells using confocal microscopy and image analysis. Prolonged cultivation of undifferentiated Caco-2 cells on transwell membranes over 21 days resulted in formation of tight monolayers and cells showed characteristics of enterocytes, like long microvilli on the cell surface and development of tight junctions.

### Internalization of QDs by Caco-2 cells

In differentiated Caco-2 cells, QD-COOH and QD-NH_2_ were associated with the membrane and could rarely be detected intracellularly after 3 days exposure as revealed by visual inspection of 3D microscopy stacks and penetration depth analysis. In comparison, both QD modifications were localized in the cell lumen of undifferentiated Caco-2 cells. The distance to the cellular membrane for QD-COOH in undifferentiated cells was measured to be −1.3 ± 0.1 µm indicating penetration into the cell lumen. The height of Caco-2 cells was estimated from image stacks to be ≥7 µm, so after uptake, QD-COOH was detected mostly in the upper part of the cells. Although uptake of QD-NH_2_ was observed by microscopy, penetration depth analysis did not indicate significant penetration of QD-NH_2_ into undifferentiated Caco-2 cells when applying a cadmium concentration of 22 µg ml^−1^. The observation of limited uptake efficiency of intact differentiated Caco-2 monolayers is consistent with existing reports [16–18]. In all these studies, TiO_2_ nanoparticles were used and aggregation of particles was observed. Song et al. used flow cytometry and TEM imaging after exposure of Caco-2 cells to 200 µg ml^−1^ TiO_2_ and found higher uptake rates in undifferentiated Caco-2 cells when compared to differentiated Caco-2 monolayers, while they also detected aggregation of TiO_2_ nanoparticles in cell medium of sizes ≥720 nm [18]. All QD modifications used in our study had a similar core height as confirmed by TEM micrographs, but under experimental conditions, agglomeration of particles was observed in microscopy studies as well, which might enhance the possibility to get trapped in microvilli of differentiated Caco-2 cells. Here, QD-NH_2_ showed more extensive agglomeration compared to QD-COOH. Similar results were obtained by Manshian et al. who found that carboxyl-QDs formed smaller agglomerates compared to amino-functionalized QDs [19].

Interestingly, penetration depth analysis revealed a broader distribution regarding the distances to the cellular membrane in undifferentiated cells when compared to differentiated cells. This might be caused by the heterogenic nature of undifferentiated Caco-2 cells prior differentiation [20]. From EM micrographs and immunostaining of the cellular surface, non microvillated and microvillated regions were observed, which might influence particle-cell interactions and uptake efficiency. On the other hand, differentiated monolayers showed a uniform surface with dense microvilli of ca. 1 µm length. These might cause a delay in uptake of particles and particle agglomerates into the cell lumen. Fisichella et al. exposed differentiated Caco-2 cells to 100 µg ml^−1^ TiO_2_ for 24 h and observed large aggregates embedded into microvilli, but no uptake was detected in TEM studies. The tendency to aggregate was also observed by DLS and sizes between 391 and 1353 nm in serum free culture medium were measured [16].

QD-PEG particles or agglomerates could neither be detected on the cell membrane in Caco-2 cells nor intracellularly after 3 days exposure to 16 nM. This might indicate loose binding and subsequent removal of QD-PEG during preparation. From drug delivery it is well known that PEGylation reduces the uptake of particles by the reticuloendothelial system (RES) resulting in increased circulation time [21, 22]. Steric hindrance and reduced adsorption of proteins on the PEGylated particle surface prevent binding to cell membranes and subsequent internalization, while length of PEG chains influence cellular uptake of nano-carriers as well [23, 24]. Kelf et al. reported reduced internalization of QDs in presence of PEG on the particle surface in three tumor cell lines [25]. QD-NH_2_, which are functionalized with PEG as well, could not be detected in undifferentiated Caco-2 using a concentration of 22 µg cadmium ml^−1^ (48 nM). On the other hand, QD-NH_2_ was internalized in undifferentiated Caco-2 cells when using 45 µg cadmium ml^−1^ (80 nM). Therefore, the particle concentration had an impact on internalization efficiency of QD-NH_2_, with a threshold concentration higher than 48 nM needed for internalization. Xiao et al., which used the same QD provided by Invitrogen, observed no difference in uptake efficiency between QD-PEG and QD-NH_2_ after 24 h exposure to mammary epithelial cell lines, but they only investigated one concentration (0.8 nM), which is much lower than the concentration we used in our study [9].

On the other hand, internalization of QD-COOH by undifferentiated Caco-2 cells was observed already at a concentration of 16 nM. Here, the surface chemistry might influence the internalization efficiency when compared to QD-NH_2_ and QD-PEG. Similar results were observed by Zhang and Monteiro-Riviere, who detected only a few CdSe/ZnS QDs coated with PEG or amine-PEG in HEK cells when using 20 nM QDs, while greater amounts of CdSe/ZnS coated with carboxyl could be seen intracellularly already at a concentration of 2 nM after 24 h exposure [11].

### Intracellular localization

After internalization of QDs by undifferentiated Caco-2 cells, the intracellular location was studied by confocal microscopy. QDs were localized in the perinuclear region of Caco-2 cells, while no uptake in the cell nucleus was observed. The cell nucleus was measured to be 7 ± 2 µm in diameter and filled-out mostly the whole cell. Perinuclear localization was reported after uptake of QDs for various cell types as well as presence of QDs within membrane bound vesicles [11, 12]. In undifferentiated Caco-cells, only a small amount of QD-COOH, but no QD-NH_2_ could be detected in EEA-1 positive endosomes after 3 days of exposure. The mean diameter of EEA-1 positive endosomes was measured to be 0.6 ± 0.1 µm, which is in accordance with previous studies reporting diameters of about 500 nm for early endosomes obtained from EM micrographs [26]. Zhang and Monteiro-Riviere investigated the mechanism of uptake of CdSe/ZnS QDs and observed a colocalization of QD-COOH with early endosome marker EEA-1 after 1 h, which gradually decreased over time [11]. Clift et al. observed partial colocalization of QD-COOH with EEA-1 in J774.A1 macrophages starting after 1 h exposure to 40 nM. Uptake of QD-NH_2_ was found to occur after 60 and 120 min, while colocalisazion of small QD-NH_2_ aggregates with EEA-1 was detected only after 2 h treatment [10].

Most studies showed that QD-COOH and QD-NH_2_, after internalization into early endosomes, are transferred into lysosomes [8, 11]. Here, the final localization of QDs in lysosomes was investigated in Caco-2 cells as well. Only QD-COOH showed partial colocalization with lamp-1 positive lysosomes while QD-NH_2_ rarely showed colocalization. In differentiated Caco-2 cells, the presence of lamp-1 positive endosomes was detected as well, but as no internalization of particles into these cells was observed no colocalization was detected. The size of lysosomes was comparable in both, undifferentiated and differentiated cells, measuring 0.9 ± 0.5 µm and 0.9 ± 0.3 µm, respectively. This resembles reports on lysosome size in mammalian cells, which were in the range of 250–1000 nm [27]. Lamp-1 positive lysosomes were significantly larger compared to EEA-1 positive endosomes in Caco-2 cells. During maturation into late endosomes and lysosomes, early endosomes increase in size due to fusion [28]. Presence of QDs in lysosomes could be underestimated due to the acid environment in these organelles (pH 4.5–5). It was shown that in acidic environment, relative fluorescence intensity of QD-COOH dilutions (7.5 nM) decreased over time [19]. In our study, fluorescence stability over 3 days was comparable between all QD modifications, but was only determined at a physiological pH of 7.4. Additionally, an endo-lysosomal escape is possible due to increased pH from protonated carboxyl groups of QDs and subsequent swelling of lysosomes [29]. From confocal microscopy images, agglomerates of QD-COOH and QD-NH_2_ were present in the lumen of undifferentiated Caco-2 cells after exposure to 45 µg cadmium ml^−1^. It has to be investigated in further studies, if they were free in the cytosol or trapped in vesicles other than EEA-1 positive endosomes or lamp-1 positive lysosomes such as multivesicular body-like structures.

### Cytotoxicity of QDs

After exposure to QDs for 3 days, no morphological changes of Caco-2 cells could be observed, even after using a concentration of 45 µg cadmium ml^−1^, which corresponds to 56 nM for QD-COOH and 80 nM for QD-NH_2_ and was therefore high compared to other studies. All QD modifications used did not induce membrane damage in undifferentiated cells after 3 days exposure, which was reflected by intact TER in differentiated Caco-2 monolayers. Using Live/Dead assay, differentiated Caco-2 cells exposed to QDs showed no increase in dead cells compared to controls. Along with composition, surface chemistry and release of free Cd^2+^ ions, toxicity of QDs depends on cell type, presence of proteins in cell medium and concentration [4, 5, 19]. Manshian et al. observed decreased viability of fibroblasts (HFF-1) exposed to concentrations higher than 15 nM QD-COOH in full serum containing medium, while epithelial cells (BEAS-2B) tolerate up to 20 nM. The epithelial cells showed highest uptake rates, yet were most resistant to cytotoxic and genotoxic effects [19]. Zhang and Monteiro-Riviere et al. detected no cytotoxicity of HEK cells exposed to 20 nM QD-COOH for 24 h, but at longer incubation for 48 h, slight cell death was noted [11]. Wang et al. observed a dose dependent cell detachment of undifferentiated Caco-2 cells exposed to CdSe/ZnS QDs functionalized with PEG-carboxyl starting at concentrations of 4.2 nM. In addition, acid treatment resulted in increased cytotoxicity due to exposure of CdSe core material [5]. It is well established that additional coating of CdSe-core QDs with a ZnS shell decreases the cytotoxic effects and prevents the release of toxic cadmium ions [4]. Mechanisms of cytotoxicity induced by QDs are discussed to be related to the production of reactive oxygen species (ROS) playing a major role by affecting cellular functions and disrupting DNA [30]. Clift et al. reported the production of ROS by measuring the glutathione levels in J774.A1 macrophages exposed for 24 h to 20, 40 and 80 nM CdSe/CdTe QD functionalized with COOH or PEG-NH_2_ and observed the modulation of intracellular Ca^2+^ signaling [31]. In our study, the formation of ROS was investigated using the H_2_DCF-DA assay. A slight but significant increase in ROS production compared to untreated cells was detected in Caco-2 cells exposed to QD-COOH using a concentration of 45 µg cadmium ml^−1^. However, interference with the assay was detected when incubating QDs in the presence of positive control SIN-1, resulting in significant increase of fluorescence intensity compared to positive control SIN-1 alone. QD dilutions in medium showed no absorption of light at 488 nm, which is the excitation wavelength of the H_2_DCF-DA assay ruling out optical interference with the test system. Kroll et al. observed significantly reduced fluorescence in presence of dispersions of carbon black, which were minimized by washing the cell monolayers prior to incubation with fluorescent DCF. In addition, they detected conversion of H_2_DCF-DA into fluorescent DCF in a cell-free assay in presence of CB dispersions [32]. This might explain the observed increase in fluorescence in our study, but has to be investigated further. Interference was also observed when using the enzyme-based CytoTox-ONE™ assay to detect membrane damage in Caco-2 cells. In the presence of QDs, the maximum fluorescence signal of cells treated with positive control Triton-X 100 was significantly reduced. Kroll et al. showed partial decrease in LDH activity induced by ZnO nanoparticles [32]. Zinc is known to inhibit the function of enzymes [33]. As Zn is also present in the shell of CdSe/ZnS QDs, release of Zn ions might be involved in the inhibition of LDH activity and subsequent reduced production of fluorescent formazan in the CytoTox-ONE™ assay. Therefore, a combination of assays utilized to investigate the cytotoxicity of QDs as well as interference controls are necessary to prevent false results.

### Transepithelial cadmium transport

Translocation of cadmium across the enterocyte-like barrier was detected, although the cell layer was intact over the exposure time as indicated by TER values higher than 250 Ω/cm^2^. However, a broad distribution in cadmium between single wells from the same treatment was observed. The retrieval of cadmium in the upper well after exposure to QDs was 95 up to 108 % of the applied concentration of 45 µg cadmium ml^−1^. In differentiated cells exposed to QD-COOH, the translocation rate was ranking from 0.1 up to 4 % of the applied cadmium concentration. In cells exposed to QD-NH_2_, translocation up to 0.6 % was detected. This is in the same range observed by Song et al. for TiO_2_ NP. They measured translocation rates of TiO_2_ NP across the Caco-2 monolayer of less than 1 % after exposure to 200 µg ml^−1^ for 24 h [18]. Janer et al. found lower translocation rates of 0.5 % when applying a concentration of 100 µg ml^−1^ of TiO_2_ NP to differentiated Caco-2 cells. Low translocation in vitro was consistent with very low absorption after oral administration in vivo [17]. In our study, no internalization of QDs in differentiated Caco-2 cells was visible in microscopy images. Geys et al. found translocation of CdSe/ZnS QDs functionalized with amino or carboxyl groups into the basal compartment only after induction of oxidative stress induced by 1 or 10 mM t-BOOH (*tert*-butyl hydroperoxide). Under these conditions, 30 % translocation over a barrier of primary rat alveolar epithelial cells was observed irrespective of the type of QDs used. This was accompanied by decreased TER values after exposure to 25 pM ml^−1^ for 24 h [34]. One explanation for the observations made in our study might be a degradation of QDs over the exposure time resulting in loss of fluorescence accompanied by agglomeration and release of cadmium. Reduction in fluorescence intensity of 30 % for both QD modifications diluted in cell culture medium was indeed observed when incubated at 37 °C and 5 % CO_2_ for up to 7 days. Koeneman et al. observed that CdTe-QD coated with hydrophilic thioglycolate capping ligands, which were maintained non-aggregated through dilution in calcium/magnesium-free phosphate buffered saline during exposure, were able to pass through the Caco-2 monolayer. When applying 10 µg cadmium ml^−1^ to the transwell membrane, 20.4 % passed through the intestinal Caco-2 barrier [35]. QD size during exposure in the study of Koeneman et al. remained 15 nm for up to 2 h and was comparable to the primary particle size of QD-COOH and QD-NH_2_ used in our study. However, in their study the integrity of the cell monolayer was impaired after exposure. In comparison, our study showed translocation without affecting the integrity of the cell layer. Mass spectra of ^114^Cd confirmed the presence of Cd ions in cell medium of the lower well after 3 days incubation on Caco-2 cells (Additional file 8), while both, Cd ions and particles were present in QD stock solutions (Additional file 9). Our results indicate that the particles are not transported via the transcellular route, but Cd ions can overcome the intact barrier.

In control experiments, a passage of cadmium across 3 µm pore-sized membranes without Caco-2 cells of 20 % and an equal distribution between upper and lower well was observed after 3 days exposure to 45 µg ml^−1^ QD-COOH. These results indicate that the used membrane size was suitable for measuring translocation rates. The pore size of the transwell membrane utilized in experiments has been described to exert influence on the translocation rate. Geys et al. showed no translocation of polystyrene latex beads (46 nm in diameter) into the basolateral compartment using 0.4 µm pore-sized membranes without cells. On the other hand, 68 % translocation of carboxyl-modified latex beads across 3 µm pore-sized membranes was observed [36]. In another study, Geys et al. evaluated the translocation of CdSe-ZnS QDs across 0.4 µm pore-sized membranes without cells and found translocation into the basolateral compartment using ICP-OES between 30 and 40 % depending on the functionalization of the QDs [34].

## Conclusion

**Fig. 15:**
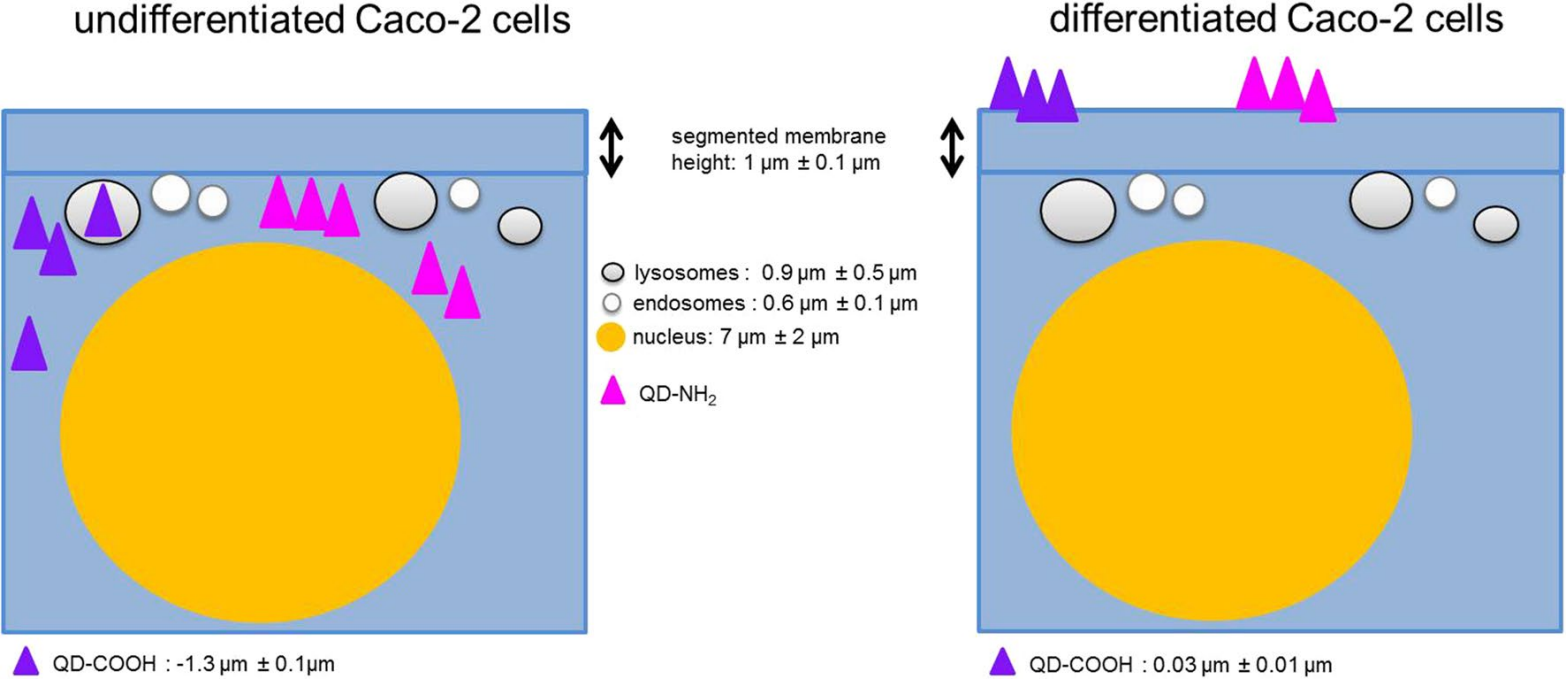
Model of QD penetration depth in Caco-2 cells. Comparison of penetration depth and localization of QD-COOH and QD-NH_2_ in undifferentiated and differentiated Caco-2 cells after 3 days exposure to 45 µg cadmium ml^−1^. In undifferentiated cells, QDs were located intracellularly and partially in lysosomes. In differentiated cells, no penetration of QDs was observed

The results showed that the internalization of CdSe/ZnS QDs depends on the differentiation status of Caco-2 cells. Differentiated, enterocyte-like monolayers of Caco-2 cells showed no internalization, independent of the surface chemistry or the QD concentration used. Membrane integrity of the barrier was not impaired by all QDs, even after long-term exposure. On the other hand, undifferentiated Caco-2 cells showed higher uptake and QDs partially localized in lysosomes. Here, internalization efficiency depends on QD concentration and surface chemistry. Figure 15 summarizes these results in a model comparing QD penetration in undifferentiated and differentiated Caco-2 cells. Using sensitive detection via ICP-MS, translocation of cadmium across the intact barrier was found. However, no internalization was visible in confocal microscopy. So combination of different detection techniques is important to get an overall view on the fate of QDs in intestine cells. Our study shows for the first time that Cd ions can overcome the intestinal barriers without affecting the integrity of the monolayer.

## Methods

### Quantum Dots (QDs)

CdSe/ZnS core/shell semiconductor nanocrystals with amino (QD-NH_2_,Qdot®655 ITK™ Amino (PEG)), carboxyl (QD-COOH, Qdot®655 ITK™ Carboxyl) and PEG (QD-PEG, Qtracker®Vascular Label) surface groups were purchased from Invitrogen. The emission maximum of all QDs was 655 nm. The concentration of QD stock solutions was 8 µM for QD-NH_2_ and QD-COOH modifications and 2 µM for QD-PEG as provided by the distributor. The cadmium concentration of a 1:1000 dispersion in water was determined using ICP-OES (Horiba Jobin Yvon Ultima 2). Prior to dilution, QD stock solutions were vortexed for 20 min followed by a short centrifugation at low rpm to remove particles from the lid of the tubes.

### Characterization of QDs

TEM micrographs of QDs were obtained by electron microscopy (Philips CM200 FEG, FEI Company, Netherlands) and were selected to estimate the height of the QDs using ImageJ software (National Institutes of Health). Dynamic light scattering (Stabisizer Nano 250, Microtrac Europe GmbH, Germany) was used to estimate the average hydrodynamic diameter of NPs and NP agglomerates dispersed in water, DPBS (Gibco) and cell culture medium. The zeta potential was measured with a Nanosizer Z (Malvern Instruments, Worcestershire, UK) in water and DPBS at 150 V using 10^−3^ M KCl as background electrolyte. In cell culture medium, the zeta potential was measured at 20 V. Each sample underwent three series of measurements (with each series comprising 40 measurements).

### Fluorescence stability of QDs

Dispersions of carboxyl and amino modified QDs were prepared in cell culture medium using a concentration of 45 µg cadmium ml^−1^ and incubated at 37 °C and 5 % CO_2_ for up to 7 days. Dispersions were vortex mixed and 100 µl were transferred into a black 96 well plate. Fluorescence was measured at excitation and emission wavelengths of 380 and 655 nm using a plate reader (infinite M 200 pro, Tecan). Average and standard deviation of fluorescence signals were calculated using four parallel samples of each QD functionalization.

### Cell culture

The Caco-2 (ACC 169) cell line was obtained from the German collection of microorganisms and cell cultures (DSMZ, Braunschweig, Germany). Cells from passage 12-26 were routinely maintained in Minimum Essential Medium (MEM), supplemented with 20 % fetal bovine serum (FBS), 2 mM L-glutamine and 1 % (v/v) non essential amino acids (all from Gibco-Invitrogen) at 37 °C and 5 % CO_2_. Caco-2 cells were grown in 75 cm^2^ plastic cultures flasks until reaching  80 % confluence and dislodged by using Cellstripper (corning).

### Exposure of cells to QDs

For confocal imaging, Caco-2 cells were seeded on transwell membrane inserts (3 µm pore size, ThinCert™, Greiner Bio-One) at a density of 10^5^ cells ml^−1^ in 12 well plates (Greiner Bio-One, Frickenhausen, Germany) for 21 days to establish differentiated cells or for 48 h to establish undifferentiated cells (80 % confluence). To determine the epithelial integrity of the differentiated cell layer, transepithelial resistance (TER) was measured before and after exposure to QDs using an EVOM (World Precision Instruments). Particle dispersions were freshly prepared in complete cell culture medium. Exposure time for all experiments was 24 h or 3 days, unless otherwise stated. For cytotoxicity experiments, Caco-2 cells were seeded in 96 well plates at a density of 10^5^ cells ml^−1^. Cells were incubated with particle dispersions at a concentration of 45 µg cadmium ml^−1^. Control samples were not exposed to QDs.

### Detection of cadmium in cell culture medium

After incubation of differentiated Caco-2 cells in presence of QD-COOH or QD-NH_2_ (45 µg cadmium ml^−1^) for 3 days, complete cell culture medium from the lower wells was collected and stored at 4 °C up to measurement. Cadmium^114^ (Cd) concentration from five samples per treatment out of two independent experiments was analyzed by inductively coupled plasma mass spectrometry (ICP-MS) in a high resolution detection mode using an ELEMENT XR (Thermo Fisher Scientific, Bremen, Germany) equipped with an auto sampler SC-E2-DX (Elemental Scientific, Omaha, USA). All calibration solutions, blind (cell culture medium) and tested samples were diluted 1:100 in MilliQ water prior ICP-MS analysis. During measurements the sample is introduced continuously into the ICP-MS system. Following nebulization, Cd^2+^ enter the plasma where they are atomized to clouds of ions resulting in a homogeneous detector signal pulse. In case of particles/agglomerates in the sample due to the high signal pulse spikes are appearing on the mass spectra (see Additional files 8 and 9). The source parameters were the following: cool gas: 16.00 L min^−1^; sample gas: 1.16 L min^−1^; Faraday deflection: −217 V; plasma power: 1250 W; peri. pump speed: 10 rpm; torch X-Pos.: 2.2 mm; torch Y-Pos.: 0.9 mm; torch Z-Pos.: −5.0 mm, at SEM voltage 1550 V. The lower limit of quantitation (LLOQ) for ^114^Cd was determined from linear regression data obtained from five calibration points prepared in blank medium in the range of 100 and 7000 ppt. The LLOQ was defined as the lowest concentration, where a precision of ≤15 % RSD was obtained (n = 3). The accuracy of ICP-MS quantification was verified by calculating the recoveries between the determined and expected ^114^Cd concentrations. Cadmium concentrations are expressed as ppb (µg L^−1^). In addition, medium of the ThinCert™ was collected after exposure and retrieval of cadmium was measured using inductively coupled plasma optical emission spectrometry (ICP-OES; Horiba Jobin Yvon Ultima 2). Prior to ICP-OES analysis, all samples were diluted 1:10 in MilliQ water. The parameters were the following: nebulizer: MicroMist, pressure: 2.05 bar, Cd: λ = 214.438 nm, flow rate: 0.7 L min^−1^.

### Immunostaining

After incubation, cells were washed two times with DPBS (Gibco) and fixed with 4 % paraformaldehyde in PBS for 30 min at room temperature. Wheat Germ Agglutinin (WGA)-Tetramethylrhodamine (W849, Invitrogen, Darmstadt, Germany) was used to stain the cell membrane and Hoechst 33342 (Invitrogen, Darmstadt, Germany) was used to stain the cell nucleus. Lamp1 (rabbit monoclonal antibody, 9091, Cell Signaling Technology) and secondary antibody Alexa Fluor 488 (goat anti-rabbit, A11008, Invitrogen, Darmstadt, Germany) were used to stain lysosomes. EEA1 (rabbit polyclonal antibody, sc-33585, Santa Cruz) and secondary antibody Alexa Fluor 488 (goat anti-rabbit, A11008, Invitrogen, Darmstadt, Germany) were used to stain early endosomes. The membrane was cut out of the ThinCert™ using a scalpel. A coverslip was placed on top of ThinCert™ membranes and mounted on glass slides with Mowiol/DABCO (Sigma Aldrich, Taufkirchen, Germany).

### Membrane integrity assays

To investigate changes in membrane integrity, CytoTox-ONE™ Homogenous Membrane Integrity Assay kit and CellTox™ Green Cytotoxicity Assay (both Promega) were utilized according to the manufacturer’s instructions. Cells incubated with medium were used as negative controls and cells treated with Triton-X 100 were used as positive controls. A no cell control was included to measure the background fluorescence of the culture medium. Fluorescence was measured at emission wavelengths of 590 nm for the CytoTox-ONE and 530 nm for CellTox Green with a Tecan Microplate reader (Molecular Devices). Interference of the used QDs with the assays was investigated prior to analysis by measuring the fluorescence of Triton-X-100 lysed cells in presence of QDs.

### ROS measurements

The production of reactive oxygen species (ROS) was measured using the probe H_2_ DCF-DA (Sigma Aldrich, Taufkirchen, Germany). H_2_DCF-DA is cell-permeable and oxidized by intracellular ROS resulting in the formation of a fluorescent probe. Caco-2 cells were seeded in black 96 well plates (10^6^ cells ml^−1^) and grown for 48 h. Further cells were washed once with pre-warmed HBSS (Hank’s buffered saline, Carl Roth GmbH, Karlsruhe) and incubated with 100 µM H_2_ DCF-DA in HBSS for 30 min at 37 °C and 5 % CO_2_. QD particle dispersions in cell culture medium were added and cells were incubated for 24 h. As positive control, 100 µM SIN-1 (Santa Cruz Biotechnology) diluted in cell culture medium was used. Fluorescence was measured at excitation and emission wavelengths of 488 and 535 nm using a Tecan Microplate Reader (Molecular Devices).

### Confocal and two-photon microscopy

A confocal laser scanning microscope Leica TCS-SP5 STED (Leica Microsystems, Mannheim, Germany) with a Leica HCX PLAN APO 100×/1.4 oil immersion objective was used to obtain image z-stacks. Specimens were imaged using the 561 nm laser line of an Argon laser for excitation of the cellular membrane label (WGA-Tetramethylrhodamine). The QDs and the cell nucleus (Hoechst 33342) were imaged using an infrared laser (MaiTai, Spectra Physics, Santa Clara, United States) running at 750 nm for two-photon excitation and detected using internal analog PMT detectors. The confocal pinhole was set to 1 AU to optimize z-sectioning in confocal mode. In two-photon mode, the pinhole was set to 3 AU to maximize photon detection. Images and z-stacks were recorded sequentially. The pixel size was set to 60 nm to avoid undersampling.

### Image processing

The first processing step was deconvolution using an iterative maximum likelihood algorithm implemented in Huygens Professional (SVI, Hilversum, Netherlands) with a theoretical PSF. For noise suppression, the QD channel was smoothed with a lateral Gaussian filter with a standard deviation of two pixels. To achieve homogeneous segmentation of the cellular membrane, the membrane channel was Gaussian filtered with a higher standard deviation of 30 pixels. No axial filtering was performed in order not to deteriorate the accuracy of axial distance measurements. In the membrane channel, a subtraction of a copy, which was axially shifted by two pixels, lead to a smaller axial extent of the segmented membrane and therefore a higher accuracy of axial distance measurements. Image analysis was performed with the “Object analyzer” tool of the Huygens software, which allows obtaining statistical information of single objects in different imaging channels. In our case, we performed axial distance measurements between the QDs and the apical cell membrane. For segmentation, the intensity threshold was automatically calculated by the default Otsu algorithm [37]. With a volume threshold of 10^5^ voxels in the membrane channel, smaller segmented membrane parts were discarded and only the main apical membrane was left. In the QD channel, falsely segmented objects below the minimal volume of a single QD were discarded by a volume threshold of 450 voxels. Objects below an intensity value of 5 % of the range between threshold and intensity maximum were discarded as well. Small QD agglomerates were meant to be separated by a watershed algorithm with a sigma of 0.1 µm for the beforehand Gaussian filter. The segmented apical membrane was set as reference object. Finally, the distances between the center of mass of the segmented QDs to the surface of the apical membrane were measured. The axial component of these distances was used for penetration depth comparison.

### Statistics

Results are presented as means and standard deviation (SD). Statistical comparisons were made with unpaired Student’s-test at a 95 % confidence level. Differences were considered significant at p ≤ 0.05.

## Additional files

10.1186/s12951-016-0222-9 Undifferentiated cells exposed to QD-PEG.Confocal images of undifferentiated Caco-2 cells exposed for three days to QD-PEG (16 nM). (A) Orthogonal views(xy, xz and yz) showing the intersection planes at the position of the yellow cross-hair. (B) Maximum intensity projection of the same z-stack. QDs (*magenta*), cell membrane (*cyan*), and nucelus (*yellow*).
10.1186/s12951-016-0222-9 Differentiated cells exposed to QD-COOH. Confocal images of differentiated Caco-2 cells exposed for three days to QD-COOH (16 nM). (A) Orthogonal views(xy, xz and yz) showing the intersection planes at the position of the yellow cross-hair. (B) Maximum intensity projection of the same z-stack. QDs (*magenta*), cell membrane (*cyan*), and nucleus (*yellow*).
10.1186/s12951-016-0222-9 Differentiated cells exposed to QD-NH_2_. Confocal images of differentiated Caco-2 cells incubated for three days with 16 nM of QD-NH_2_. (A) Orthogonalviews (xy, xz and yz) showing the intersection planes at the position of the yellow cross-hair. (B) Maximum intensity projection of the same z-stack. QDs (*magenta*), cell membrane (*cyan*), and nucleus (*yellow*).
10.1186/s12951-016-0222-9 Undifferentiated Caco-2 cells exposed to cell culture medium without QDs (ctr). Confocal images of undifferentiated Caco-2 cells exposed only to cell culture medium. (A) Orthogonal views (xy, xzand yz) showing the intersection planes at the position of the yellow cross-hair. (B) Maximum intensity projection ofthe same z-stack. Cell membrane (*cyan*) and nucleus (*yellow*).
10.1186/s12951-016-0222-9 Differentiated Caco-2 cells exposed to cell culture medium without QDs (ctr). Confocal images of differentiated Caco-2 cells exposed only to cell culture medium. (A) Orthogonal views (xy, xzand yz) showing the intersection planes at the position of the yellow cross-hair. (B) Maximum intensity projection ofthe same z-stack. Cell membrane (*cyan*) and nucleus (*yellow*).
10.1186/s12951-016-0222-9 Membrane integrity measurements using CytoTox-ONE™ Assay. Membrane integrity of undifferentiated Caco-2 cells was measured after exposure to QD-COOH and QD-NH_2_ (16nM) for 24 h. Error bars represent SD of 3 independent experiments. In the presence of QDs, the fluorescence intensity of the positive control decreased. ** significantly different from positive control, p ≤ 0.01.
10.1186/s12951-016-0222-9 Measurement of ROS. Undifferentiated Caco-2 cell were exposed for 24 h to QD-COOH at a concentration of 45 µ cadmium ml^−1^ SIN-1was used as a positive control to induce the production of ROS. In the presence of QDs, the fluorescence intensity of the positive control increased. * significantly different from ctr, p ≤ 0.05,** Significantly different from positive control, p ≤ 0.01.
10.1186/s12951-016-0222-9 Mass spectra of measured ^114^Cd. During measurements the sample, an aqueous solution, is introduced continuously into the ICP-MS system. Following nebulization, Cd^2+^ enter the plasma where they areatomized to cloud of ions resulting in a homogeneous detector signal pulse. Mass spectra of the sample (C) is identical to ionic ^114^Cd content in water (A) and medium (B).
10.1186/s12951-016-0222-9 Mass spectra of measured ^114^Cd in stock solutions. During measurements the sample, anaqueous solution, is introduced continuously into the ICP-MS system. In case of particles/agglomerates in thesample due to the high signal pulse spikes are appearing on the mass spectraVisible spikes on the mass spectra (D) and (E) illustrate the presence of particles/agglomerates in QD stocksolutions (red circles).

## Abbreviations

QD: quantum dots
Cd: cadmium
PEG: polyethylene glycol
COOH: carboxyl group
NH_2_: amino group
ICP-MS: inductively coupled plasma mass spectrometry
ICP-OES: inductively coupled plasma optical emission spectrometry
LLOQ: lower limit of quantification
TER: transepithelial resistance
MEM: minimal Essential Medium
TEM: transmission electron microscopy
ZO-1: zonula occludens
EEA-1: early endosome antigen
WGA: wheat germ agglutinin
SIN-1: 3-morpholinosydnonimine
H_2_DCF-DA: 2’,7’-dichlordihydrofluorescin-diacetat
ROS: reactive oxygen species
DLS: dynamic light scattering
