# Supplementary figures and images for: Penetration of CdSe/ZnS quantum dots into differentiated vs undifferentiated Caco-2 cells
Source: J Nanobiotechnology. 2016 Sep 26;14:70. doi: 10.1186/s12951-016-0222-9 (PMC5037864; doi:10.1186/s12951-016-0222-9)

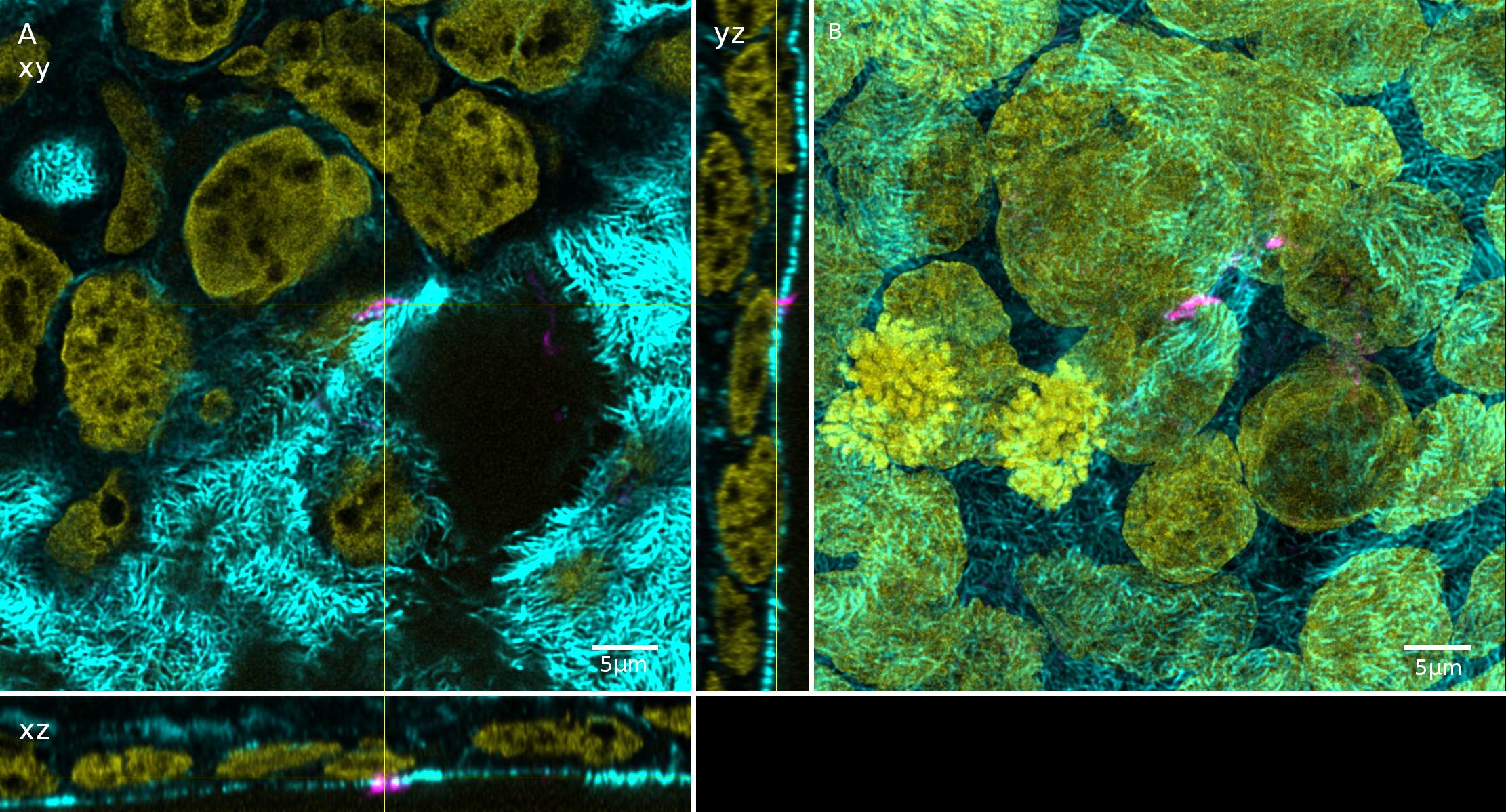

Supplement: Supplementary file 1 — 10.1186/s12951-016-0222-9 Undifferentiated cells exposed to QD-PEG.Confocal images of undifferentiated Caco-2 cells exposed for three days to QD-PEG (16 nM). (A) Orthogonal views(xy, xz and yz) showing the intersection planes at the position of the yellow cross-hair. (B) Maximum intensity projection of the same z-stack. QDs (magenta), cell membrane (cyan), and nucelus (yellow). [file 12951_2016_222_MOESM1_ESM.jpg]

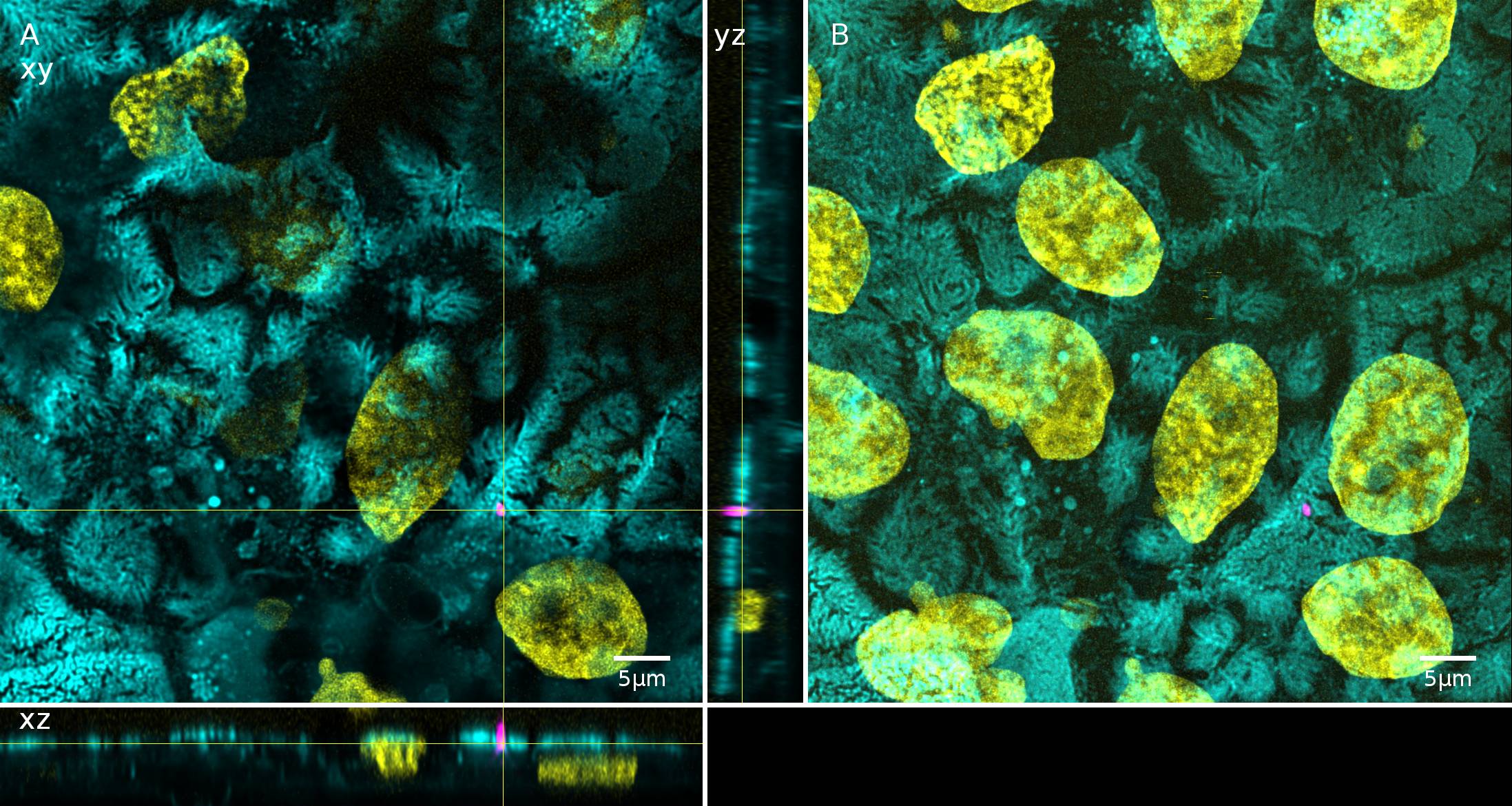

Supplement: Supplementary file 2 — 10.1186/s12951-016-0222-9 Differentiated cells exposed to QD-COOH. Confocal images of differentiated Caco-2 cells exposed for three days to QD-COOH (16 nM). (A) Orthogonal views(xy, xz and yz) showing the intersection planes at the position of the yellow cross-hair. (B) Maximum intensity projection of the same z-stack. QDs (magenta), cell membrane (cyan), and nucleus (yellow). [file 12951_2016_222_MOESM2_ESM.jpg]

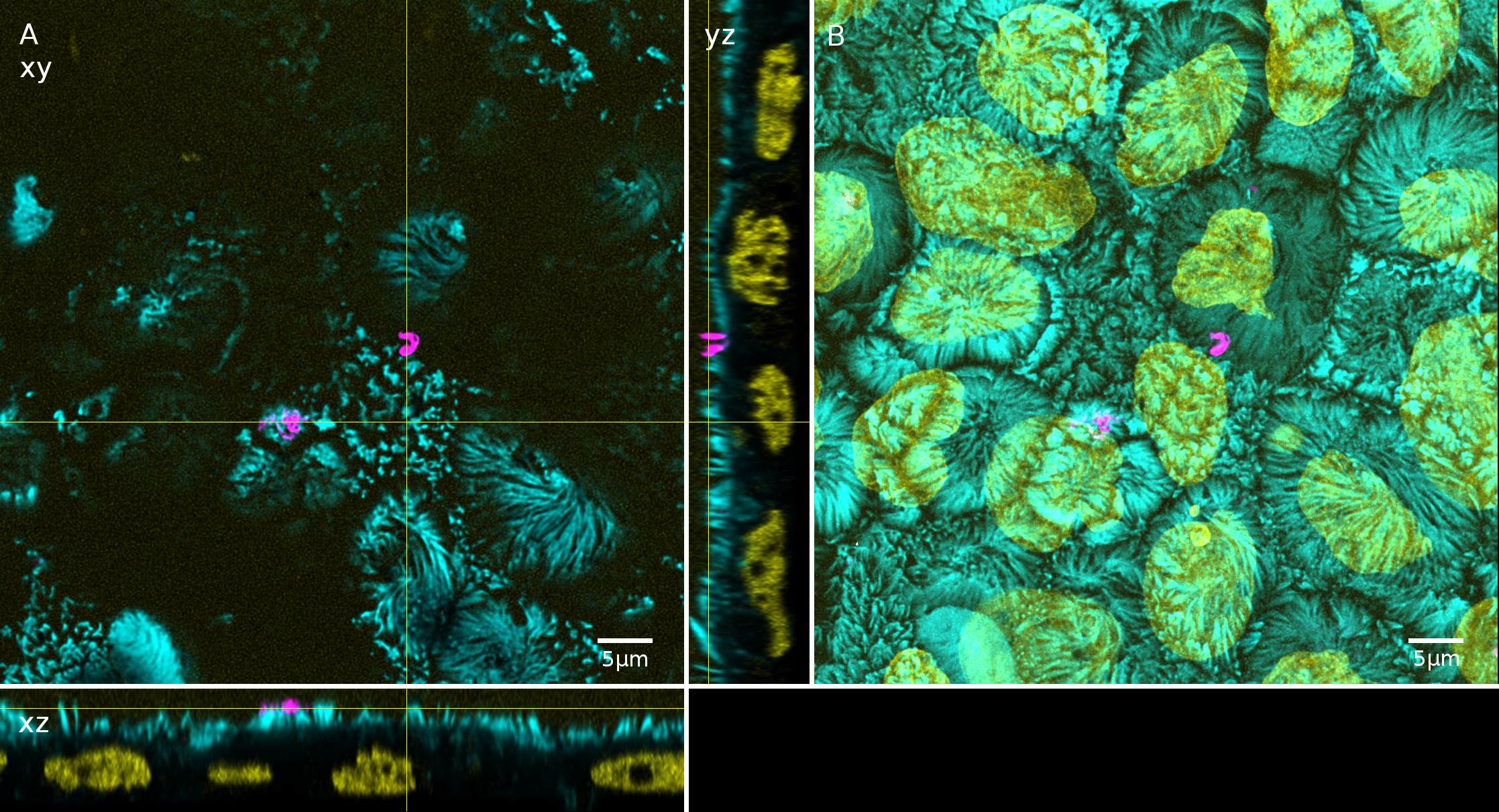

Supplement: Supplementary file 3 — 10.1186/s12951-016-0222-9 Differentiated cells exposed to QD-NH2. Confocal images of differentiated Caco-2 cells incubated for three days with 16 nM of QD-NH2. (A) Orthogonalviews (xy, xz and yz) showing the intersection planes at the position of the yellow cross-hair. (B) Maximum intensity projection of the same z-stack. QDs (magenta), cell membrane (cyan), and nucleus (yellow). [file 12951_2016_222_MOESM3_ESM.jpg]

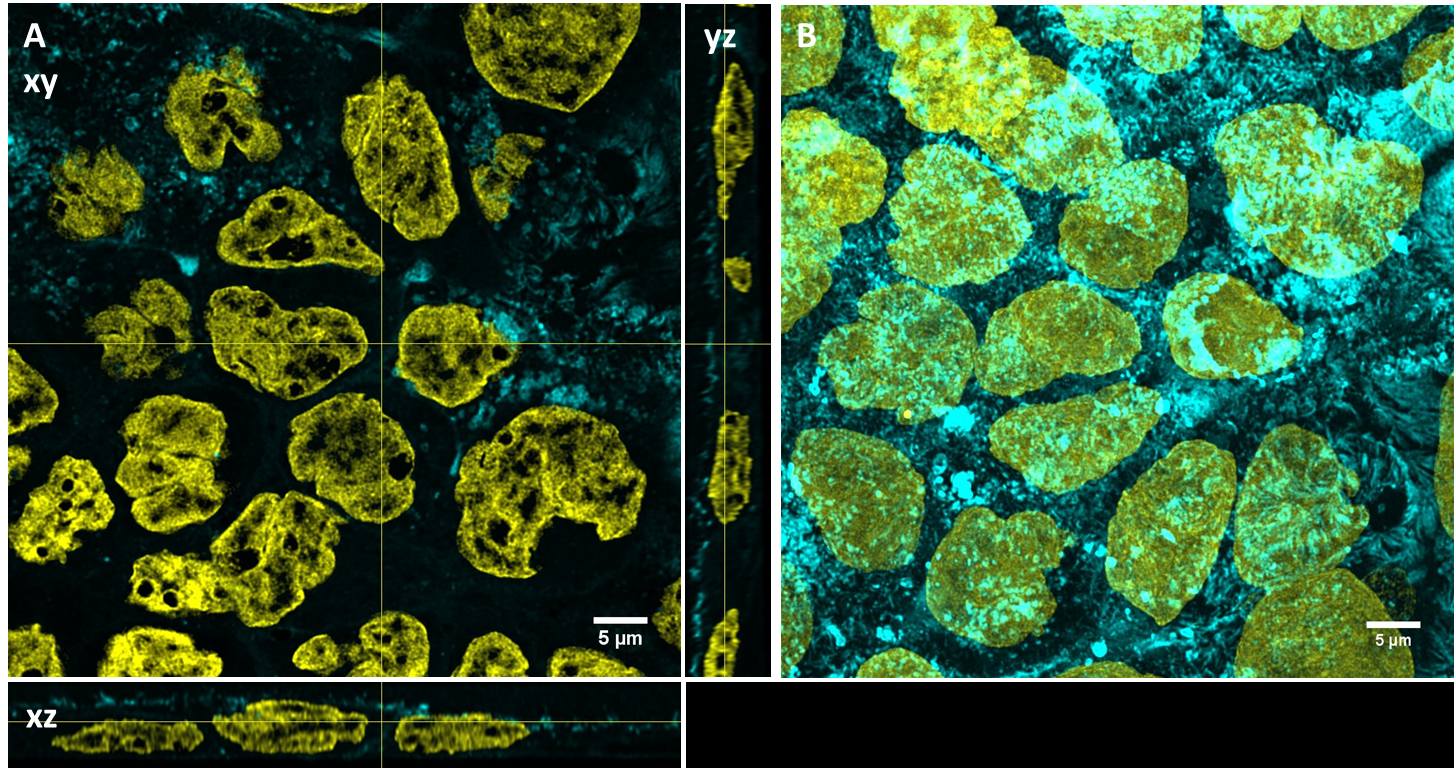

Supplement: Supplementary file 4 — 10.1186/s12951-016-0222-9 Undifferentiated Caco-2 cells exposed to cell culture medium without QDs (ctr). Confocal images of undifferentiated Caco-2 cells exposed only to cell culture medium. (A) Orthogonal views (xy, xzand yz) showing the intersection planes at the position of the yellow cross-hair. (B) Maximum intensity projection ofthe same z-stack. Cell membrane (cyan) and nucleus (yellow). [file 12951_2016_222_MOESM4_ESM.png]

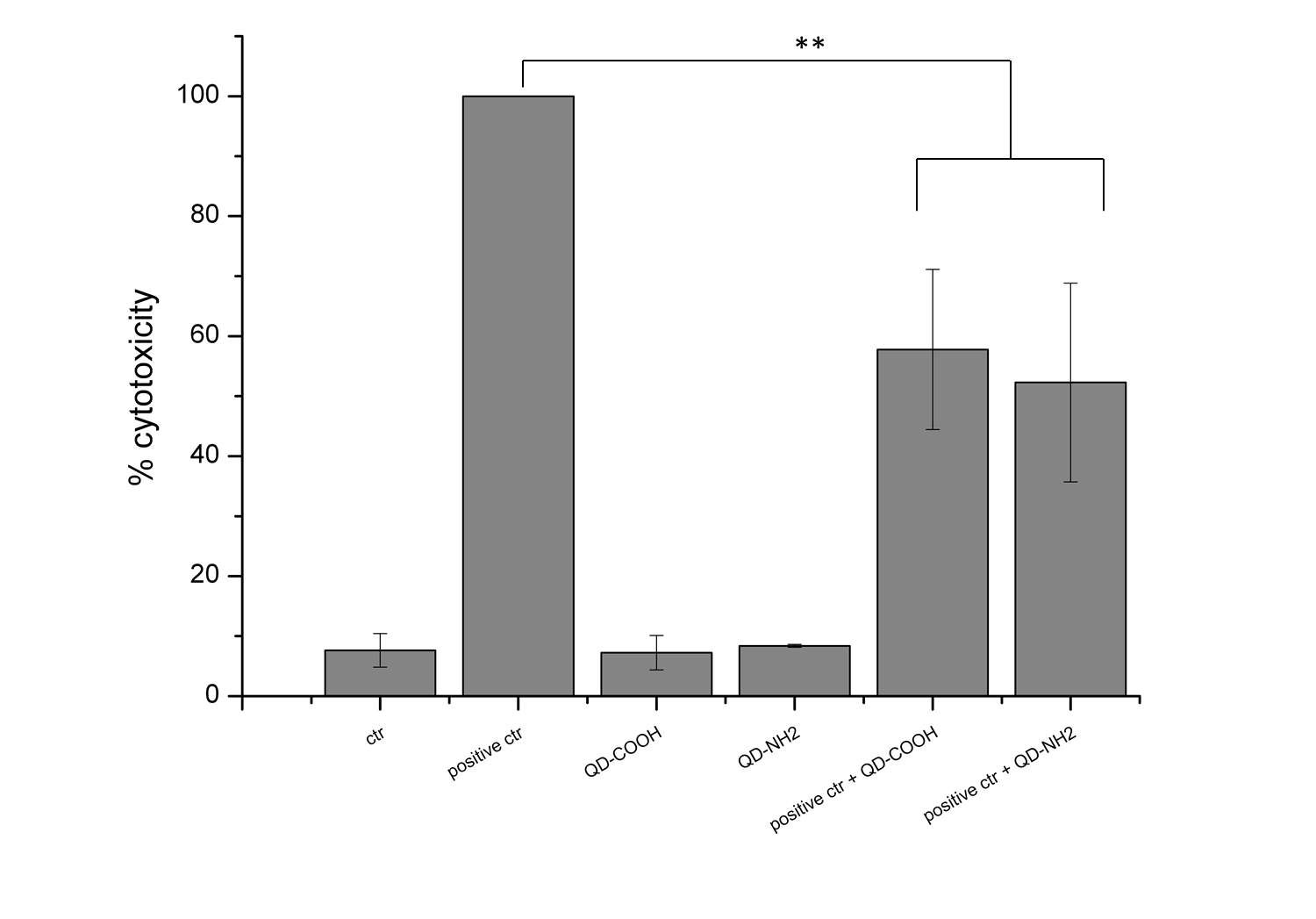

Supplement: Supplementary file 6 — 10.1186/s12951-016-0222-9 Membrane integrity measurements using CytoTox-ONE™ Assay. Membrane integrity of undifferentiated Caco-2 cells was measured after exposure to QD-COOH and QD-NH2 (16nM) for 24 h. Error bars represent SD of 3 independent experiments. In the presence of QDs, the fluorescence intensity of the positive control decreased. ** significantly different from positive control, p ≤ 0.01. [file 12951_2016_222_MOESM6_ESM.png]

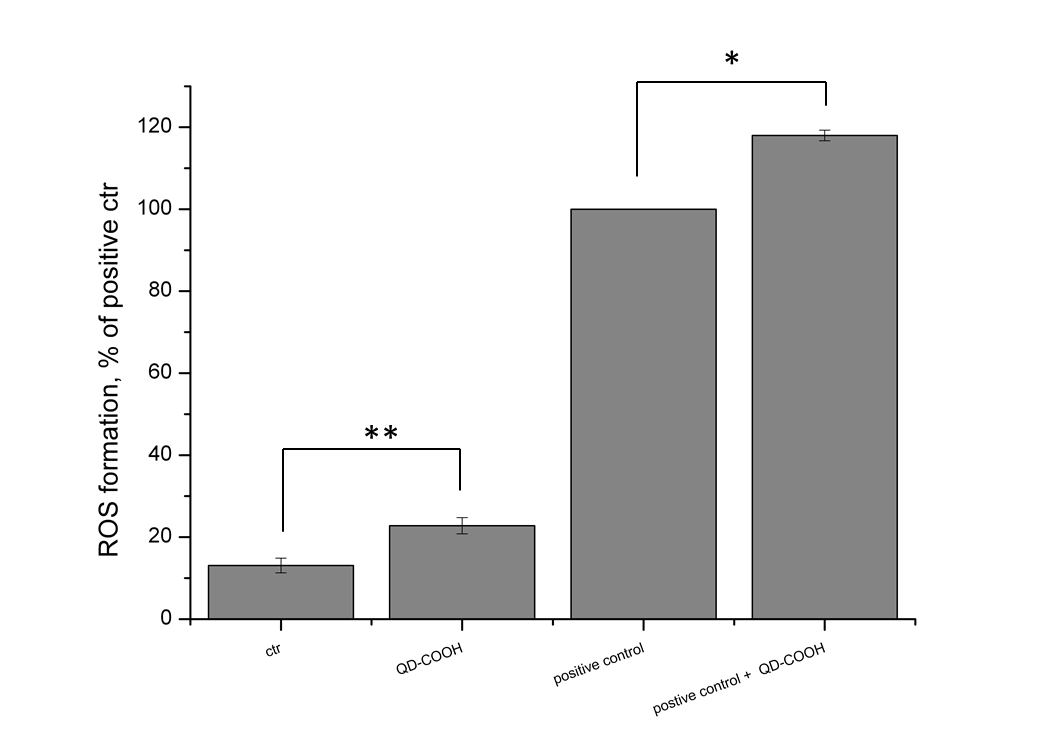

Supplement: Supplementary file 7 — 10.1186/s12951-016-0222-9 Measurement of ROS. Undifferentiated Caco-2 cell were exposed for 24 h to QD-COOH at a concentration of 45 µ cadmium ml−1 SIN-1was used as a positive control to induce the production of ROS. In the presence of QDs, the fluorescence intensity of the positive control increased. * significantly different from ctr, p ≤ 0.05,** Significantly different from positive control, p ≤ 0.01. [file 12951_2016_222_MOESM7_ESM.png]

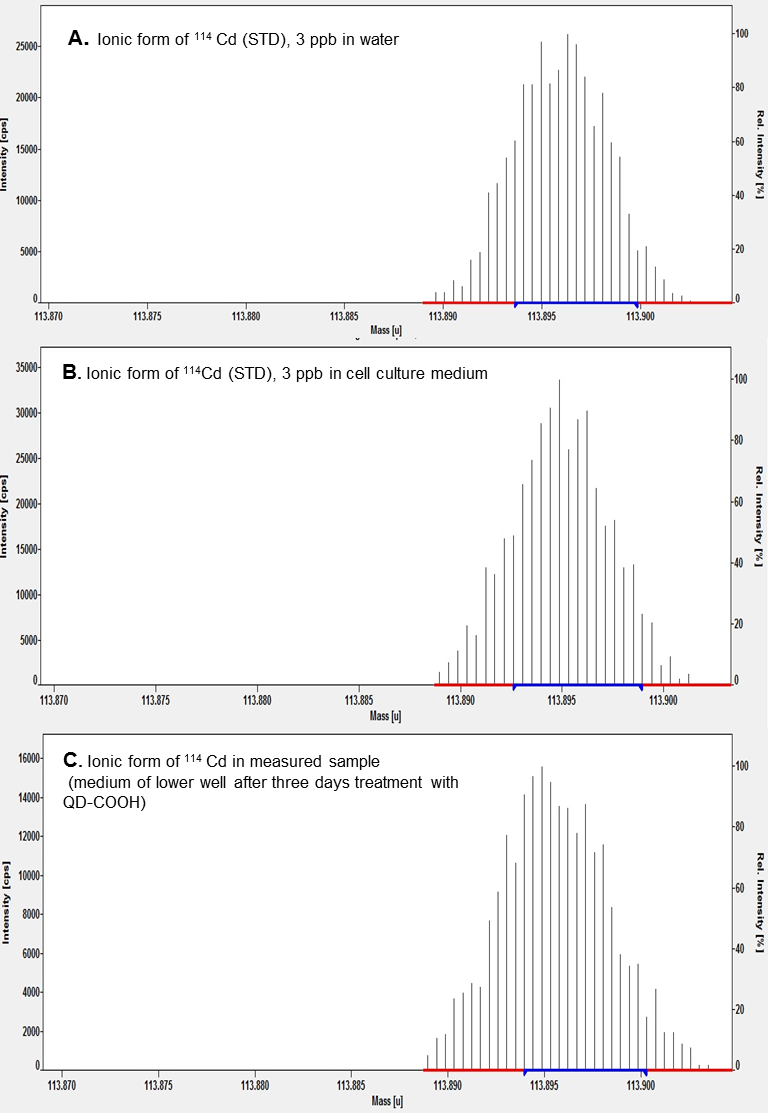

Supplement: Supplementary file 8 — 10.1186/s12951-016-0222-9 Mass spectra of measured 114Cd. During measurements the sample, an aqueous solution, is introduced continuously into the ICP-MS system. Following nebulization, Cd2+ enter the plasma where they areatomized to cloud of ions resulting in a homogeneous detector signal pulse. Mass spectra of the sample (C) is identical to ionic 114Cd content in water (A) and medium (B). [file 12951_2016_222_MOESM8_ESM.png]

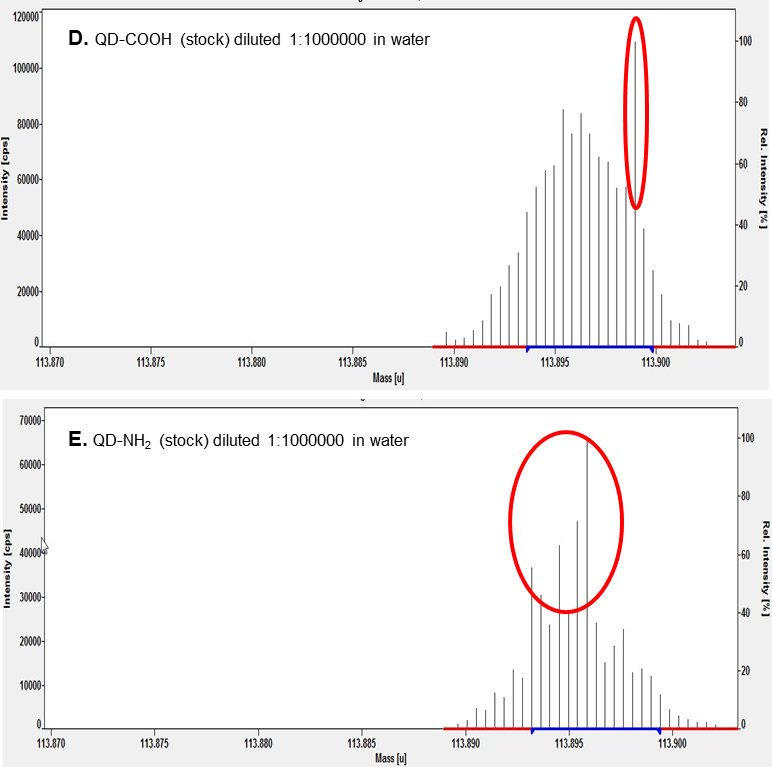

Supplement: Supplementary file 9 — 10.1186/s12951-016-0222-9 Mass spectra of measured 114Cd in stock solutions. During measurements the sample, anaqueous solution, is introduced continuously into the ICP-MS system. In case of particles/agglomerates in thesample due to the high signal pulse spikes are appearing on the mass spectraVisible spikes on the mass spectra (D) and (E) illustrate the presence of particles/agglomerates in QD stocksolutions (red circles). [file 12951_2016_222_MOESM9_ESM.png]
